# How children learn to understand language meanings: a neural model of adult–child multimodal interactions in real-time

**DOI:** 10.3389/fpsyg.2023.1216479

**Published:** 2023-08-03

**Authors:** Stephen Grossberg

**Affiliations:** Center for Adaptive Systems, Boston University, Boston, MA, United States

**Keywords:** neural network, visual perception, language development, circular reaction, meaning, joint attention, learning, object recognition

## Abstract

This article describes a biological neural network model that can be used to explain how children learn to understand language meanings about the perceptual and affective events that they consciously experience. This kind of learning often occurs when a child interacts with an adult teacher to learn language meanings about events that they experience together. Multiple types of self-organizing brain processes are involved in learning language meanings, including processes that control conscious visual perception, joint attention, object learning and conscious recognition, cognitive working memory, cognitive planning, emotion, cognitive-emotional interactions, volition, and goal-oriented actions. The article shows how all of these brain processes interact to enable the learning of language meanings to occur. The article also contrasts these human capabilities with AI models such as ChatGPT. The current model is called the ChatSOME model, where SOME abbreviates Self-Organizing MEaning.

“The child…projects the whole of his verbal thought into things.”

Jean Piaget

“The most complex type of behavior that I know: the logical and orderly arrangement of thought and action.”

Karl Lashley

## 1. Introduction: toward understanding how children and adults learn language meanings

This article proposes a functional and mechanistic analysis of key brain processes that enable a child to learn language utterances and their meaning. Learning the meaning of language utterances allows children and adults to describe and understand their perceptual and emotional experiences in the world, and to generate appropriate actions based on this understanding. Such learning typically begins when a baby who knows no language interacts with someone who does, often a parent or other caregiver.

The current article proposes how multiple brain regions interact to support the learning of language utterances and their meanings. This explanation builds upon biological neural network models of how our brains make our minds that have been getting steadily developing during the past half-century. These models provide principled and unifying explanations of hundreds of psychological and neurobiological experiments. Many model predictions have also been confirmed by subsequent experiments. A self-contained and non-technical exposition that provides an overview of this progress is described in Grossberg (2021).

The importance of how language learning and its meaning occur for understanding the human condition is reflected by a large number of articles and books that have been written about it (e.g., Boroditsky, 2000; Boroditsky and Ramscar, 2002; Kable et al., 2002, 2005; Richardson et al., 2003; Hauk and Pulvemuller, 2004; Gallese and Lakoff, 2005; Pecher and Zwaan, 2005; Wallentin et al., 2005, 2011; Gibbs, 2006; Zwaan and Taylor, 2006; Chatterjee, 2008, 2010; Fischer and Zwaan, 2008; Holtgraves and Kashima, 2008; Kemmerer et al., 2008; Mahon and Caramazza, 2008; Boulenger et al., 2009; Dove, 2009; Raposo et al., 2009; Casasanto and Dijkstra, 2010; Glenberg, 2010; Glenberg and Kaschak, 2010; Saygin et al., 2010; Bergen, 2012; Aziz-Aadeh, 2013; Fernardino et al., 2013; Watson et al., 2013; Colston, 2019; Carey, 2022; Gleitman and Gleitman, 2022). Although such contributions include many facts about language learning and meaning, they do not provide a unifying mechanistic neural explanation of these data, or of the organizational principles that shape these brain mechanisms.

This article models brain processes of language learning and meaning in *real-time*; notably, how these processes unfold moment-by-moment as the learning individual interacts with the world. Language learning and meaning are emergent properties of widespread brain interactions operating in real time. No available experimental method can provide a mechanistic link between such interactive brain dynamics and the emergent behavioral properties that they cause. A rigorous mechanistic theory is needed to bridge this explanatory gap.

The article will discuss how a baby first learns, during visual and auditory *circular reactions*, to look at, or point to, objects of interest, as well as how to produce, store, and learn simple speech sounds that create a foundation for learning to imitate the language utterances of its teachers.

Before a baby or child can learn language from an adult, it must first be able to pay attention to, and learn to recognize, that adult's behaviors, notably to pay attention to and recognize an adult caregiver's face when he or she is speaking. To accomplish this feat, several additional learning processes need to occur.

Learning to recognize an adult caregiver's face includes the ability to recognize multiple views of the face as experienced from multiple positions, orientations, and distances. In other words, a young child effortlessly learns to solve the *invariant pattern recognition problem*! This learning process includes the learning of facial views while a baby looks up at its mother as it suckles milk from her breast or a bottle.

The learning of invariant object representations coexists with the learning of individual views of the object. In addition to enabling the child to recognize its mother's face from multiple perspectives, this ability enables the child to also learn that specific views of her face correlate with her looking, pointing, or otherwise acting on objects in a given direction. These invariant and view-specific representations reciprocally interact with each other via learned connections so that a child can invariantly recognize its mother's face even as it uses her currently perceived view to predict her ongoing actions.

Section 3 will discuss how learning of invariant object categories such as a face requires interactions between *spatial attention* in the dorsal, or Where, cortical processing stream with *object attention* in the ventral, or What, cortical processing stream, notably between cortical areas PPC (posterior parietal cortex) and IT (inferotemporal cortex), respectively. More generally, all the learning that is important for a child's understanding and survival requires interactions between multiple brain regions.

Another important example of inter-region interactions during learning occurs when the cognitive representations for learned object recognition interact reciprocally via learned connections with emotional representations. These cognitive-emotional interactions, which include brain regions such as the prefrontal/temporal cortices and amygdala/hypothalamus, amplify the cognitive representations of currently valued objects in a scene. These cognitive-emotional interactions will be discussed in Section 4.

Competitive interactions among object representations in the temporal and prefrontal cortices can choose the currently most valued objects in a scene that may be cluttered with multiple other objects. It is explained in Section 4 how these motivationally amplified representations successfully compete for spatial attention to attract an observer's gaze, such as when a child orients to look at its mother's face. The child can then begin to learn how current views of her face predict where she is looking, pointing, or otherwise acting.

A prerequisite for learning to understand and produce simple language utterances is for a sequence of speech sounds, or items, to be stored temporarily in a *working memory* that is designed to support learning and stable memory of stored phonemes, syllables, words, and sentences. Such working memories occur in multiple regions of the prefrontal cortex. Reciprocal interactions occur between sequences of stored working memory items and the learned speech categories, or *list chunks*, that categorize the sequences. Bottom-up interactions from the working memory to the list chunk level enable the list chunks to be learned. Top-down interactions from an active list chunk to the working memory can dynamically stabilize the learning of the item sequence that it codes. How and why working memories and their learned list chunks have the designs that they do will be explained in Section 14.

During language recall, list chunks read-out into working memory their learned item sequences. *Volitional signals*, or GO signals, from the basal ganglia enable a sequence that is stored in working memory to be performed in the correct order and at the desired speed. This role for the basal ganglia will be discussed in Section 15. Also, how more complex rhythmic performances, as during the singing of musical lyrics and melodies, can be achieved using *spectral timing circuits* in the cerebellum and basal ganglia is discussed.

When these capabilities interact in real time, a child can learn that specific language phrases and sentences strongly correlate with specific visual objects and events that the child is simultaneously watching a teacher use or perform. In this way, a child can learn that a phrase like “mommy walks” correlates with a simultaneous percept of the described action. The phrase hereby acquires meaning by representing this perceptual experience.

Learning of auditory language utterances occurs in the ventral, or What, cortical processing stream for object attention and categorization, including the temporal and prefrontal cortices (Rauschecker and Scott, 2009; Kimppa et al., 2015; Sections 14 and 19) Learning to visually recognize mommy also occurs in the What stream, including the inferotemporal cortices (Tanaka et al., 1991; Tanaka, 1996).

In contrast, the perceptual representation of mommy walking includes representations in the dorsal, or Where cortical stream for spatial attention and action, including cortical areas MT and MST. Learning language meanings thus often requires What-to-Where cortical stream interactions that link language utterances to the perceptual experiences that they describe. Sections 4 and 5 will discuss how learning between these What and Where stream interactions occurs.

Mommy can also activate affective representations, which enable a baby or child to have feelings about mommy. As noted above, midbrain regions such as the amygdala and hypothalamus help to generate such feelings when they interact reciprocally with perceptual and cognitive representations that represent mommy. Section 4 will discuss how interactions between cognitive and emotional representations are learned and how they focus motivated attention upon a valued caregiver like mommy.

Language meaning is thus embodied in the interactions between a language utterance and the perceptual and affective experiences with which it is correlated, much as the word “mommy” can activate a complex set of learned associations that prime perceptual, cognitive, emotional, and action representations that have been experienced during previous interactions with her. When the word “mommy” is internally heard as a child thinks about mommy, the activation of this language representation can prime multiple perceptual and affective representations. Volitional GO signals from the basal ganglia (Section 15) can then activate internal visual experiences such as visual imagery and felt emotions, thereby enabling a child or adult to understand the perceptual and affective meaning of “mommy” without needing to observe her.

The OpenAI project ChatGPT (GPT, Generative Pre-trained Transformer) has shown a remarkable ability to provide useful answers in often impressive language about a broad number of topics, as well as sometimes surprising failures to do so. ChatGPT has acquired this ability after having been fed immense language databases by human operators, as well as a probabilistic algorithm to predict what comes next in its currently active linguistic context (http://chat.openai.com; Newport, 2023).

In contrast to the biological neural network models that are described below, ChatGPT cannot do fast incremental learning with self-stabilizing memories about novel situations that include unexpected events. In particular, it cannot rapidly learn about the rare events that are often the basis of great discoveries, such as being able to learn the first breakout of a new disease in an environment where lots of people are sick with other diseases, or to make a great scientific discovery based on just a few key facts combined with a deep experimental intuition and a creative imagination. ChatGPT cannot learn anything after its parameters are frozen before it is used in applications.

ChatGPT would not seem nearly as impressive if it were not being interpreted by humans who do know the real-world meaning of the language that they use, including ChatGPT creators and users.

Perhaps most importantly, and central to the main theme of the current article, ChatGPT does not know the real-world meaning of its predictions.

The current article describes brain processes that enable humans to learn a language that expresses the meaning of perceptually and affectively experienced events in the real world. I call the neural model that accomplishes this ChatSOME (SOME = Self-Organizing MEaning).

## 2. Building upon visual and auditory circular reactions

**Figure 1:**
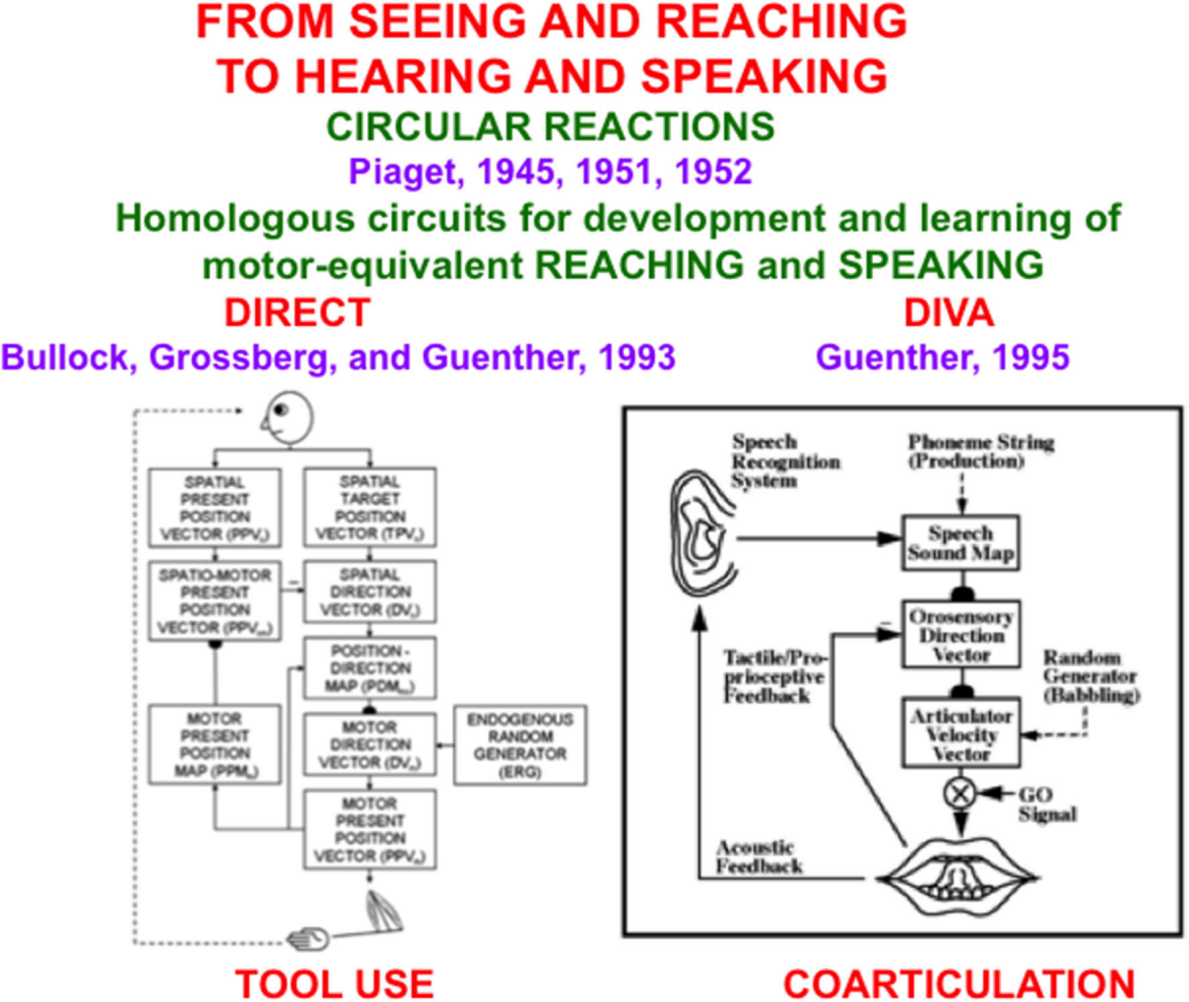
The DIRECT and DIVA models have homologous circuits to learn and control motor-equivalent reaching **(Left)** and speaking **(Right)**, with tool use within the hand-arm system, and coarticulation within the speech articulator system, resulting properties [Reprinted with permission from Grossberg (2021)].

Early steps in language development prepare a learned scaffold upon which later learning of language meanings can build. These processes are reviewed by Grossberg (2021). As noted there, perception-cognition-action *circular reactions* occur during auditory and visual development, to whose understanding the pioneering Swiss clinical and developmental psychologist Jean Piaget has richly contributed (e.g., Piaget, 1945, 1951, 1952). Recent reviews and discussions of Piaget's work include those of Singer and Revenson (1997), Carey et al. (2015), and Burman (2021). In particular, all babies normally go through a *babbling phase* during which a *circular reaction* can be learned (Figure 1).

During a *visual* circular reaction, babies endogenously babble, or spontaneously generate, hand/arm movements to multiple positions around their bodies. Babbled movements endogenously sample the workspace within which a baby can reach. As their hands move in front of them, their eyes reactively look at their hands. While the baby's eyes are looking at its hands, an associative map is learned from its hand positions to the corresponding eye positions, *and* from its eye positions to hand positions. The learned map between eye and hand in both directions is the “circular” reaction.

After map learning occurs, when a baby, child, or adult looks at a target position with its eyes, this eye position can use the learned associative map to prime the activation of a movement command to reach the target position in space. If a volitional GO signal (Figure 1, Right), or “the will to act”, is also activated by opening the correct basal ganglia gate, then the chosen target position is fully activated and enables a hand/arm movement to reach the foveated position in space, as when a baby looks at her toes and then moves her hands to hold them. Because our bodies grow for many years as we develop from babies into children, teenagers, and adults, these maps continue updating their learned parameters to enable control of accurate movements using our current bodies and limbs.

An *auditory* circular reaction occurs during its babbling phase. During an auditory circular reaction, babies endogenously babble simple sounds that sweep out the workspace of sounds that they can create. The babies also hear the sounds that they create. When the motor commands that caused the sounds and the auditory representations of the heard sounds are simultaneously active in the baby's brain, a map is learned between these auditory representations and the motor commands that produced them.

After enough map learning occurs, a child can use the map to approximately imitate sounds from adult speakers. It can then incrementally learn how to speak using increasingly complicated speech and language utterances, again under volitional control.

Several biological neural network models have been developed to explain how visual circular reactions enable reaching behaviors to be learned (Bullock and Grossberg, 1988, 1991; Gaudiano and Grossberg, 1991, 1992; Bullock et al., 1993, 1998), and auditory circular reactions enable speech and language behaviors to be learned (Grossberg, 1986; Grossberg and Stone, 1986; Cohen et al., 1988; Guenther, 1995; Guenther et al., 2006). Grossberg (2021) summaries solutions of the multiple design problems that must be solved by each brain for these processes to work well, including neural models that solve these design problems, and both psychological and neurobiological data that support these models.

These processes enable babies to learn to imitate simple sentences that adult caregivers say, such as “Mommy walk”, “Mommy throw ball”, and so on.

## 3. Learning invariant categories of valued objects: Where-to-What stream interactions

Before a child can learn to say anything about, or to, mommy, it must be able to notice that mommy is there by paying attention to her. To explain this, I will review brain processes that contribute to social cognition (Grossberg and Vladusich, 2010; Grossberg, 2021).

**Figure 2:**
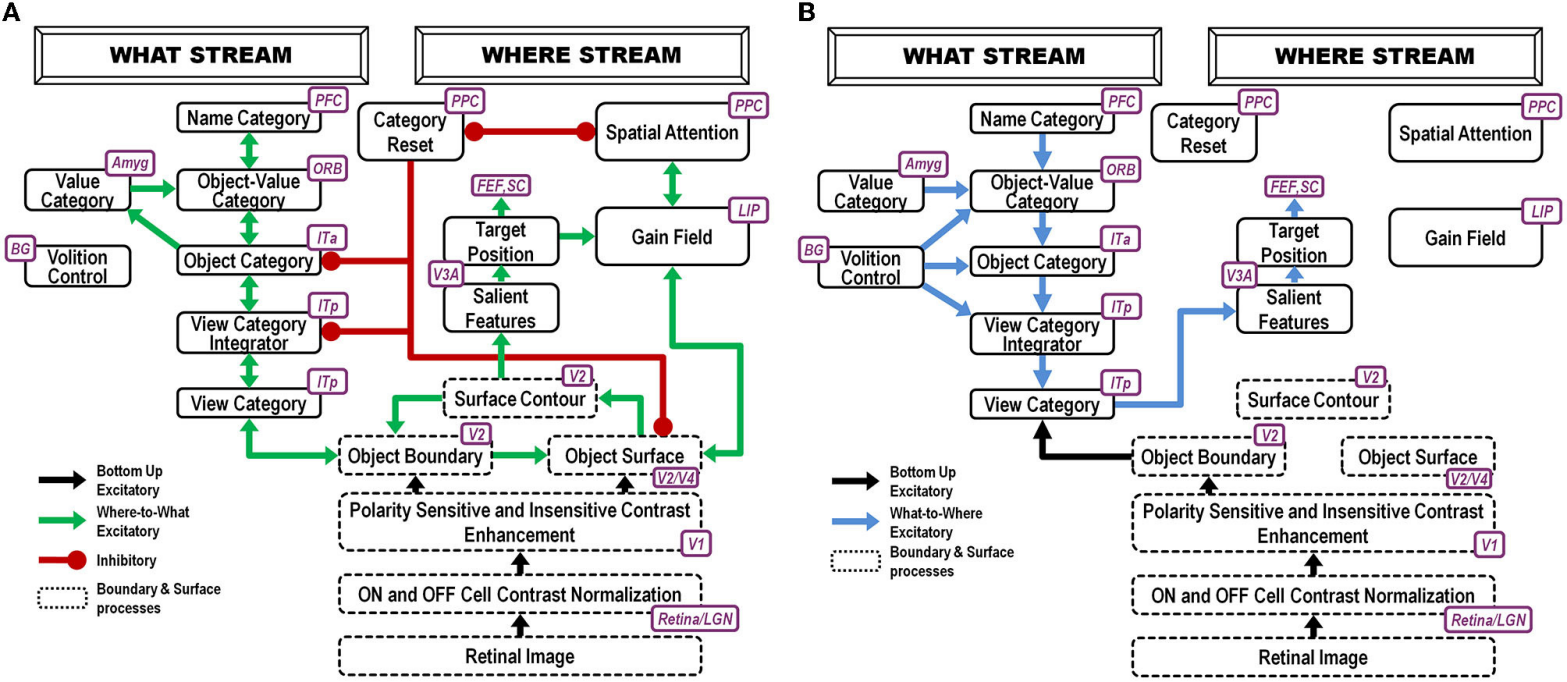
ARTSCAN Search model (Chang et al., 2014) macrocircuit: **(A)** These interactions of the ARTSCAN Search model enable it to learn to recognize and name invariant object categories. Interactions between spatial attention in the Where cortical stream, via surface-shroud resonances, and object attention in the What cortical stream, via feature-category resonances that obey the ART Matching Rule, coordinate these learning, recognition, and naming processes **(B)**. The ARTSCAN Search model can also search for a desired target object in a scene [Reprinted with permission from Grossberg (2021)].

Before a child can pay attention to mommy, it must first learn to recognize her face using both an invariant object category representation as well as category representations of specific facial views. The ARTSCAN Search model proposes how this happens (Figure 2).

Such category learning can begin when the baby looks up at mommy's face, say during suckling or other close encounters where mommy provides high-value primary rewards. The baby then gradually learns view-specific category representations of her face in the posterior inferotemporal cortex, or ITp, as well as an invariant category representation of it in the anterior inferotemporal cortex, or ITa. The learned associations between ITp and ITa are bidirectional (Figure 2A), as during all object learning processes that are capable of dynamically stabilizing their learned memories, as modeled by Adaptive Resonance Theory, or ART (Grossberg, 1978a, 2019, 2021; Carpenter et al., 1989; Grossberg and Wyse, 1992; Fazl et al., 2009; Cao et al., 2011; Grossberg et al., 2011b,c, 2014; Chang et al., 2014; Section 21).

The bottom-up pathways from ITp to ITa are an adaptive filter for learning the invariant category's adaptive weights. The adaptive weights in the top-down pathways from ITa to ITp learn expectations that focus attention on the critical features that are used to recognize object views in ITp. The reciprocal excitatory interactions between ITp and ITa trigger a *category-to-category resonance* that dynamically stabilizes the memories that are learned by adaptive weights in both the bottom-up and top-down pathways. ART is said to solve the *stability-plasticity dilemma* because it can support fast incremental learning of such categories without experiencing catastrophic forgetting.

An invariant object representation in ITa is learned by being associated with multiple view-specific representations that are learned in ITp. This is proposed to happen as follows: The first ITp representation to be learned activates an initially uncommitted cell population in ITa that will become the invariant category representation. To become an invariant object representation, this chosen cell population in ITa must remain active while multiple view-specific representations in ITp are associated with it as the observer inspects the object.

Each view-specific category in ITp is inhibited, or reset (see category reset stage in Figure 2), when the next one is activated, learned, and associated with the emerging invariant category in ITa. Why is the ITa representation *not* reset when this happens, since it was originally activated by the first ITp representation to be reset? This is explained by how the child's *spatial attention* sustains its focus on mommy's face with a *surface-shroud resonance* that prevents reset of the ITa representation while the baby attends multiple views of mommy's face (Fazl et al., 2009). An active shroud inhibits the category reset processing stage that would otherwise have inhibited ITa. Each ITp view-specific category that is learned can then be associated with the persistently active ITa category, thereby converting it into an *invariant* object category.

An *attentional shroud* is spatial attention that fits itself to the shape of the object surface that is being attended (Tyler and Kontsevich, 1995). When spatial attention shifts to another object, the category reset stage is disinhibited and learning of a new invariant category can begin.

A surface-shroud resonance is depicted in Figure 2A between visual cortical area V4 (lumped with V2 as V2/V4 in the figure) and the posterior parietal cortex, or PPC. A *gain field* in the lateral intraparietal area, or LIP, occurs between these cortical areas to carry out the change of coordinates from retinotopic surface coordinates of the attended object's view-specific categories to head-centered spatial attention coordinates of the invariant category.

ITp-ITa category learning goes on in the ventral, or What, cortical processing stream. Modulation of ITa learning by an attentional shroud is due to Where-to-What stream signals that occur while a surface-shroud resonance is active between the attended object's surface representation in cortical area V4 of the What stream and the shroud in the posterior parietal cortex, or PPC, of the dorsal, or Where, stream.

In summary, a surface-shroud resonance between V4 and PPC sustains spatial attention on mommy's face during invariant category learning between ITp and ITa. Recurrent inhibitory interactions occur within these cortical regions to select the most predictive cell representations, focus attention on them, and suppress outlying features (Figure 2A).

While a surface-shroud resonance modulates invariant category learning in the What stream, it also supports *conscious seeing* of the surface representation in V4 upon which PPC maintains spatial attention.

Figure 2B shows the brain regions whereby a search can be carried out for a desired target object in a cluttered scene, thereby solving the Where's Waldo problem (Chang et al., 2014).

Supportive psychophysical and neurobiological data are reviewed in Grossberg (2021), whose Chapter 3 explains how boundaries and surfaces interact to generate percepts of rounded shapes like a ball.

## 4. Cognitive-emotional interactions focus motivated attention on a valued caregiver's face

A baby can learn its mommy's face while she is engaged in actions that reward the baby, notably feeding it with her breast or a bottle. The milk, warmth, comfort, happiness, etc. that are experienced during feeding are all positively rewarding. As category learning occurs, categories that persist over time, notably the emerging invariant face category, are bidirectionally associated with positive emotional centers, also called *drive representations* or *value categories*, that are activated in the baby's brain by mommy's rewarding activities. These drive representations are in the amygdala/hypothalamic system.

**Figure 3:**
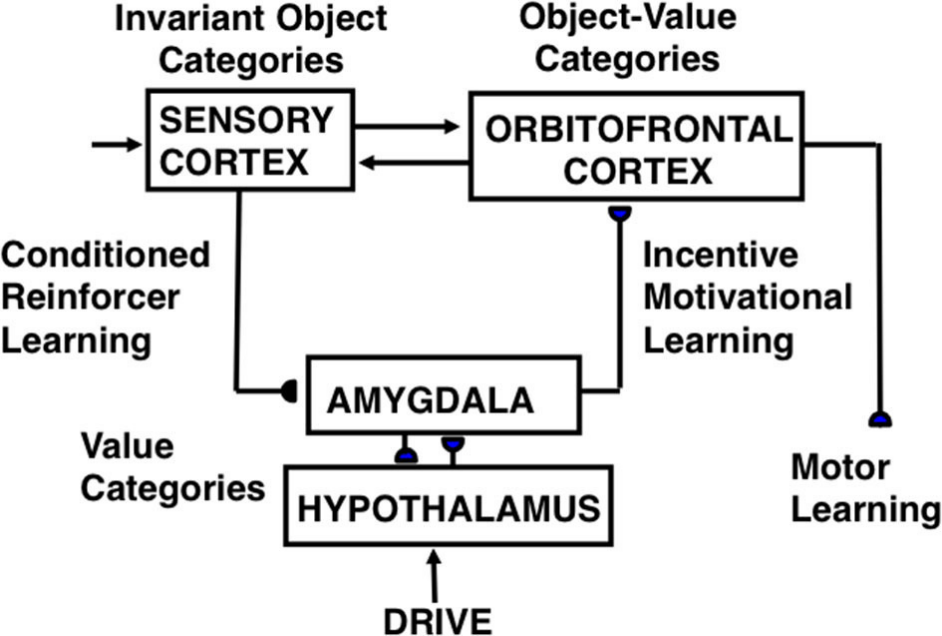
Macrocircuit of the functional stages and anatomical interpretations of the Cognitive-Emotional-Motor, or CogEM, model [Reprinted with permission from Grossberg (2021)].

A neural model of *cognitive-emotional resonances*, called the Cognitive-Emotional-Motor, or CogEM (Figure 3), has been incrementally developed to achieve an ever-broadening interdisciplinary explanatory range (Grossberg, 1971a,b, 1972a,b, 1974, 1975, 1978b, 1982, 1984a,b, 2018, 2019; Grossberg and Levine, 1987; Grossberg and Schmajuk, 1987, 1989; Fiala et al., 1996; Dranias et al., 2008; Grossberg et al., 2008; Chang et al., 2014; Franklin and Grossberg, 2017). C*ognitive-emotional resonance* links attended objects to feelings. Its positive feedback loop associates an invariant object category with an active drive representation. The positive feedback loop generates conscious feelings about the attended object while maintaining motivated attention on the object and reading-out commands for actions that realize currently valued goals.

After category learning and cognitive-emotional learning occur, when the baby and its mommy are in different spatial locations, the baby's attention is drawn to whatever familiar view of mommy's face is seen. If an unfamiliar view is similar to a familiar one, then the most similar familiar view category is activated.

Whereas invariant category learning requires a Where-to-What interaction between cortical streams, *orienting* to a valued familiar face requires a What-to-Where stream interaction (Figure 2B). Such an interaction is needed because the motivationally amplified *invariant* face category in ITa, being positionally invariant, cannot directly control orienting to a particular position in space.

However, the invariant ITa category of mommy's face is amplified by cognitive-emotional feedback. The invariant category can then *prime* all the ITp view-specific categories of mommy's face (Figure 2A). Bottom-up inputs from a current view of mommy's face (green arrow from the object's boundary representation in Figure 2A) determine which ITp category will fire.

Such an ITp category is both view-specific *and* positionally specific. It can, in turn, trigger reciprocal excitatory interactions with the surface representation in cortical area V4 of the object view that it categorizes (Figure 2A). When this surface representation is amplified, it triggers a surface-shroud resonance that most strongly activates the object's position via spatial attention in PPC, while inhibiting other positions via recurrent inhibition across PPC. PPC can then command looking, reaching, and other actions directed toward the position of mommy's face.

These interactions have been modeled by the ARTSCAN Search model (Chang et al., 2014). Figure 2A provides a block diagram of the Where-to-What and What-to-Where stream interactions for learning invariant object recognition categories. Figure 2B shows how categories that are currently valued can control actions to look at and acquire them.

## 5. Joint attention: how looking at a valued face triggers learned orienting to an attended object

How does a baby learn to associate an attended view of mommy's face with the position in space where she is looking or pointing? (Tomasello and Farrar, 1986; Emery et al., 1997; Deák et al., 2000; Frischen et al., 2007; Materna et al., 2008). For starters, as mommy moves her arm, and perhaps her body too, to point to something for the baby to look at, spatial attention can flow from mommy's attended face representation along her arm to her hand.

### 5.1. G-waves: long-range apparent motion helps to track attended moving objects

**Figure 4:**
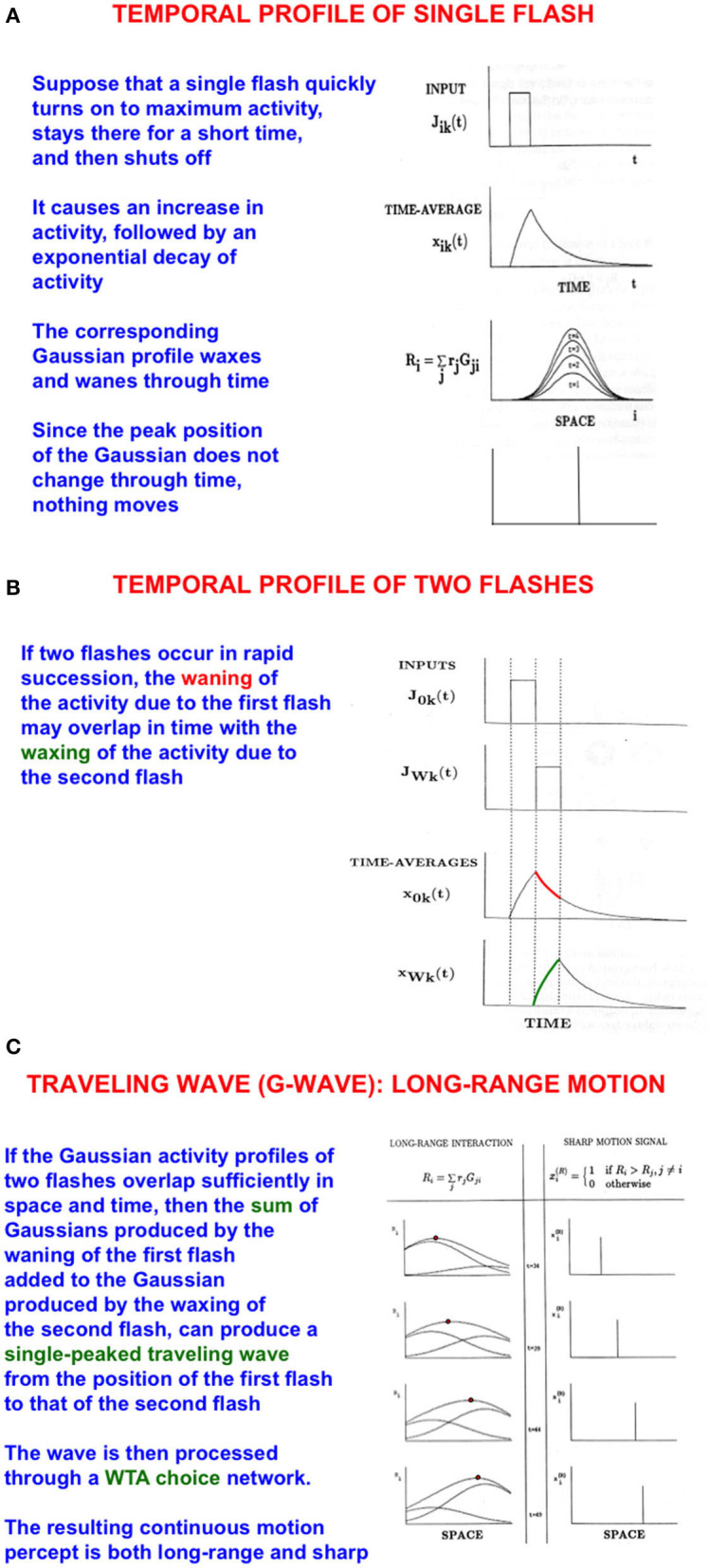
Long-range apparent motion: **(A)** As a flash waxes and wanes through time, so too do the activities of the cells in its Gaussian receptive field. Because the maximum of each Gaussian occurs at the same position, nothing is perceived to move. **(B)** If two flashes occur in succession, then the cell activation that is caused by the first one can be waning while the activation due to the second one is waxing. **(C)** The sum of the waning Gaussian activity profile due to the first flash and the waxing Gaussian activity profile due to the second flash has a maximum that moves like a traveling wave from the first to the second flash [Adapted with permission from Grossberg (2021)].

I call such a flow of spatial attention a G-wave, or Gauss-wave (Figures 4A–C), because it describes how attention smoothly flows along successive positions on mommy's body, starting with an attended initial position and ending at a moving target position. I discovered G-waves to model psychophysical data about *long-range apparent motion* (Grossberg and Rudd, 1989, 1992; Francis and Grossberg, 1996a,b; Grossberg, 2014).

The simplest example of long-range apparent motion occurs when two flashes occur at different positions at successive times. Within a range of spatial separations and temporal delays, observers perceive a smooth motion from the first flash to the second. This visual illusion is called *beta motion* (Exner, 1875; Kolers, 1972). Long-range apparent motion is not just a laboratory curiosity. Many motion percepts that are important for survival are long-range apparent motion percepts in naturalistic settings, such as continuous tracking of a prey or predator as it darts behind successive bushes or trees at variable speeds. G-waves also exhibit these properties, including flowing *behind* occluding objects to maintain attention on the moving target (Grossberg, 2021).

### 5.2. Learning an association from a view of mommy's face to the position of her hand in space

As noted above, a G-wave can travel from mommy's face to her hand as she points at an object of interest. An association can then be learned from the view-specific category of mommy's face to the final position of her hand in space. The TELOS model simulates how such an association is learned (Brown et al., 2004). This view-specific category can, during subsequent experiences, use the learned association to predict where mommy is looking and thus to enable a baby or child to look in the direction that mommy is looking, whether or not she is pointing there.

**Figure 5:**
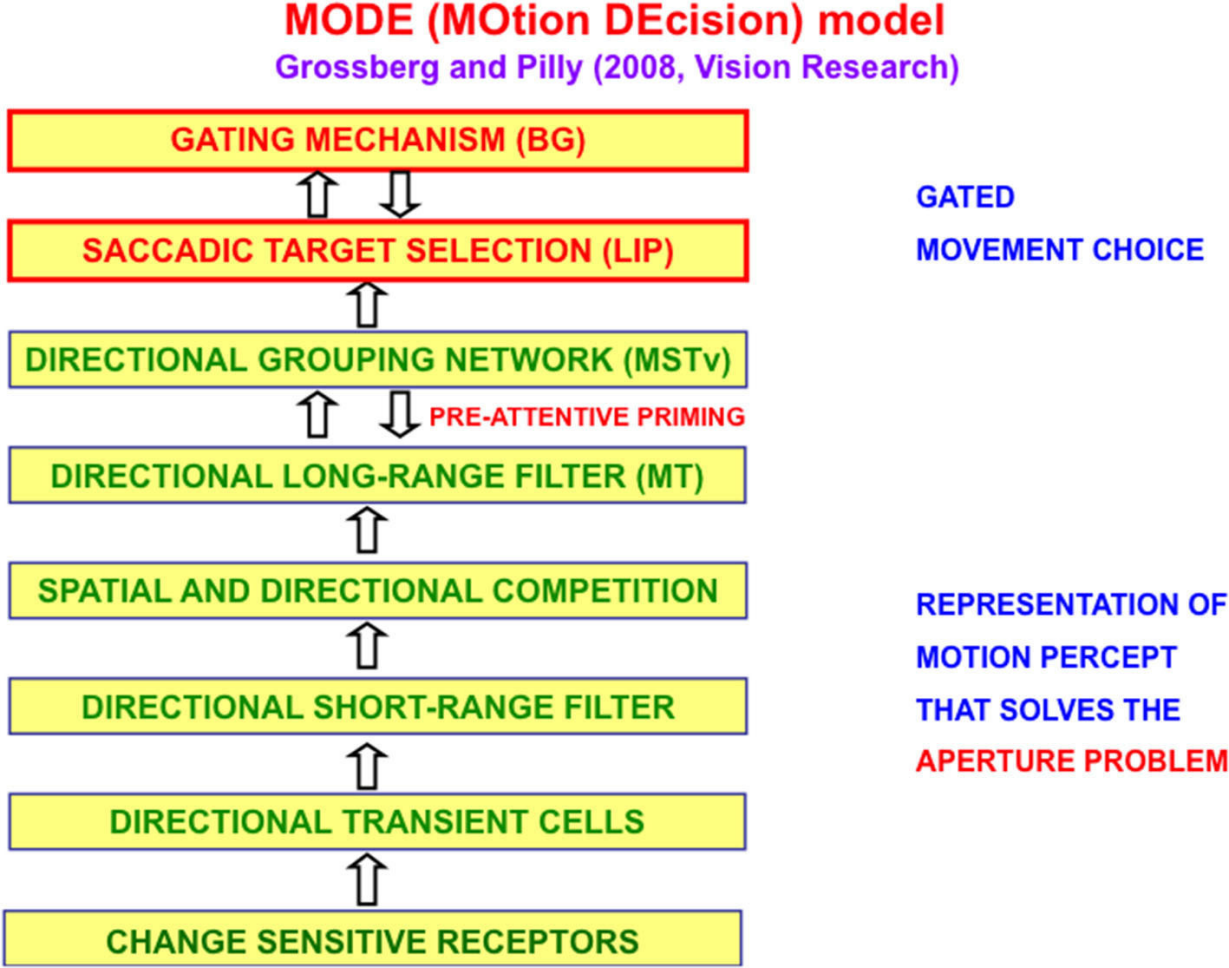
The MODE model (Grossberg and Pilly, 2008) uses motion preprocessing stages, collectively called the Motion BCS [green letters], as its front end, followed by a saccadic target selection circuit in the model LIP region [red letters] that converts motion directions into movement directions. These movement choices are also under basal ganglia (BG) control. MT, Middle Temporal area; MSTv, ventral Middle Superior Temporal area; LIP, Lateral Intra-Parietal area [Reprinted with permission from Grossberg (2021)].

The MOtion DEcision, or MODE, model of Grossberg and Pilly (2008) defines and simulates on the computer the motion processing stages that compute the direction of object motion and convert it into saccadic eye movements that maintain fixation upon a moving target. Figure 5 summarizes the multiple processing stages in the Where stream whose interactions accomplish this.

**Figure 6:**
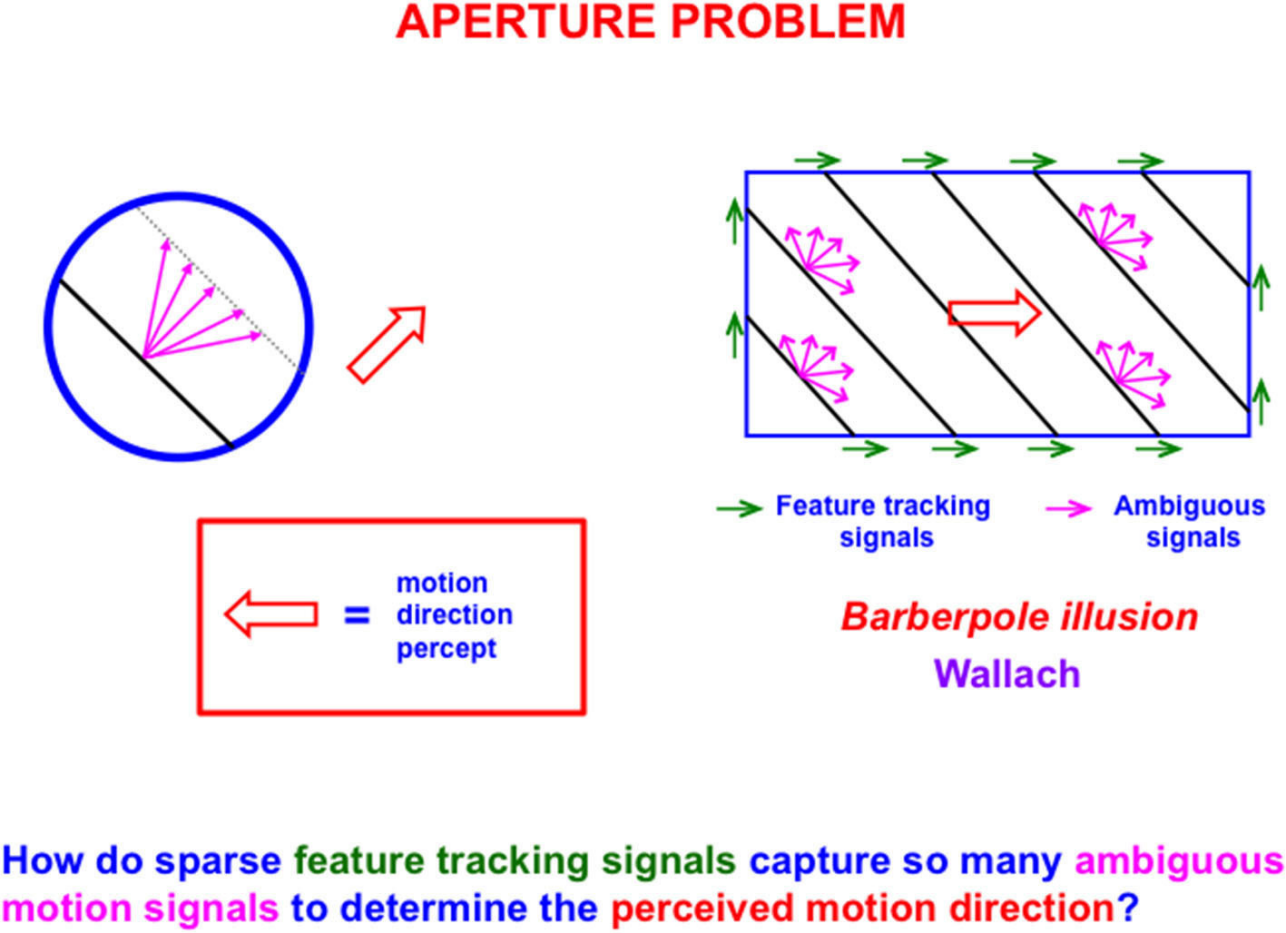
The perceived direction of an object is derived either from a small subset of feature tracking signals (green arrows), or by voting among ambiguous signals when feature tracking signals are not available (purple arrows), to determine an estimate of object motion direction and speed (outline red arrow) [Reprinted with permission from Grossberg (2021)].

The first problem that MODE solves is the *aperture problem* (Guilford, 1929; Wallach, 1935 [English translation by Wuerger et al., 1996]), whereby our brains transform directionally ambiguous motion information received by our retinas into a representation of an object's motion direction and speed. Figure 6 illustrates this problem. Wallach (1935) noted that a moving line that is seen within a circular aperture always looks like it is moving in a direction perpendicular to its orientation, no matter what its real motion direction is. That is because all local motion signals are ambiguous (purple arrows). In a rectangular aperture, *feature tracking signals* (green arrows) define a consistent direction. MODE chooses this consistent motion direction while suppressing ambiguous directions. This choice process is called *motion capture* (Grossberg, 2021).

MODE also converts the direction of object motion into a command in that direction. When a target unpredictably changes its direction and speed of motion, additional interacting brain regions coordinate saccadic and smooth pursuit eye movements to maintain fixation (Grossberg et al., 2012).

## 6. Learning to associate seeing mommy with her name: building on circular reactions

As learning of mommy's invariant face category stabilizes, the baby can learn to associate it with an auditory production of mommy's name. This ability also requires a Piagetian *circular reaction*. As noted in Section 2, this auditory circular reaction occurs during its own babbling phase (Figure 1). During it, babies endogenously babble the simple sounds that they can create. The babies also hear the sounds that they create via auditory feedback. The auditory representations of the heard sounds are associated with the motor commands that caused these sounds.

A child uses the learned map to approximately imitate sounds that they hear from adult speakers. Their approximations of adult sounds may initially be coarse, but with the help of adult feedback and passive hearing of other speakers' utterances, the map is incrementally refined, leading to adult speech and language utterances.

As in the case of the visual circular reaction for looking and reaching, the auditory circular reaction for speaking is also under volitional control by the basal ganglia.

If mommy responds positively to hearing her name called, the child's resultant feelings can trigger cognitive-emotional interactions that strengthen the learned association between seeing mommy and saying mommy.

## 7. Learning categories of mommy's movements: illuminants, boundaries, surfaces, and rebounds

Before a child can learn a short sentences such as “mommy points” or “mommy walks”, the child must first learn to recognize her movements and learn names for them. Suppose that a child sees one side of mommy as she walks, how does the child's brain represent any object's motion? How does it represent the motion of a complex form like a human body?

Section 5 reviewed how a G-wave can track a moving object, such as mommy's hand as she points. It can also track mommy's moving body. Before an extended object like a body can be tracked, however, the baby's brain needs to preprocess the visual signals that it receives on its retinas from the object to create a sufficiently complete and stable representation of her body that can be tracked.

Objects are often seen in multiple lighting conditions. A baby needs to compensate for them by *discounting the illuminant* to prevent confusion of object form with ever-changing conditions of object illumination. Discounting the illuminant is accomplished by a recurrent on-center off-surround network whose neurons obey the membrane equations of neurophysiology, also called shunting interactions (Grossberg, 1988; Grossberg and Todorovic, 1988; Hong and Grossberg, 2004; Grossberg and Hong, 2006). Such a *recurrent competitive field* (Grossberg, 2013) occurs at multiple stages of neuronal processing, including the retina (Grossberg, 1987a,b; Grossberg and Hong, 2006).

After discounting the illuminant, multiple interacting cortical processing stages complete the *boundary* and *surface* representations that are used to recognize mommy. Boundaries and surfaces are incomplete when registered by a retina because retinas contain a *blind spot* where signals from retinal photodetectors send their signals along axons to form the optic nerve. The blind spot is as large as the fovea, but we never see it. The retina is also covered by nourishing veins. These occlusions are also not seen (Kolb, H. Webvision: The Organization of the Retina and Visual System: http://webvision.med.utah.edu/book/part-i-foundations/simple-anatomy-of-the-retina/).

**Figure 7:**
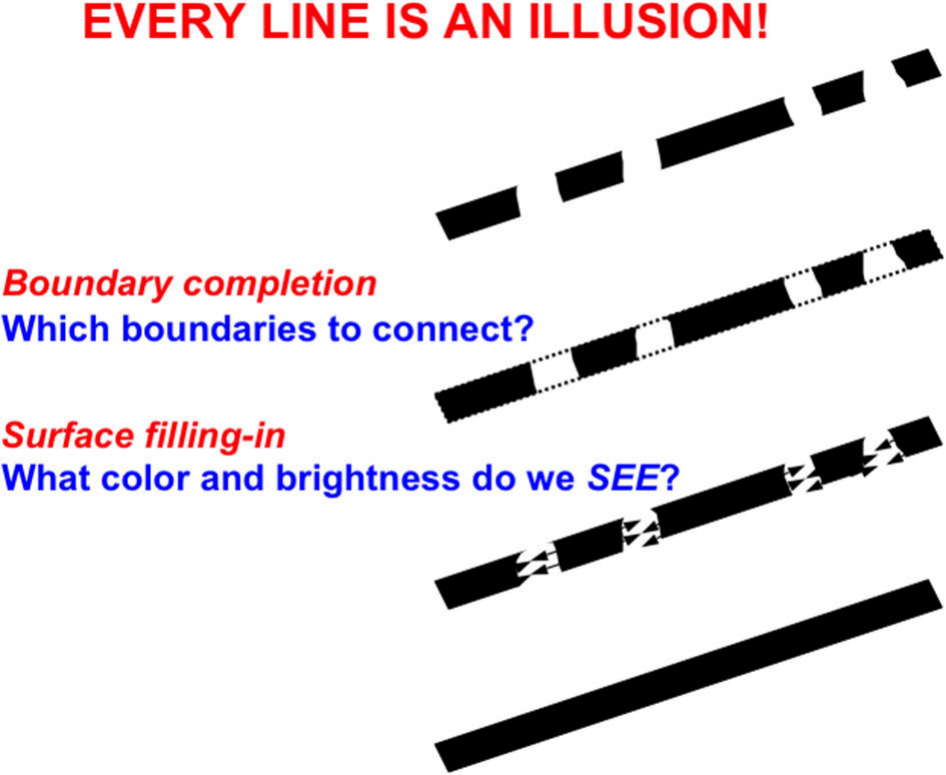
“Every line is an illusion” because regions of the line that are occluded by the blind spot or retinal veins (top figure: the occluded retinal input is a series of black colinear fragments) are completed at higher levels of brain processing by boundary completion (second figure down: dotted lines indicate boundary completion) and surface filling-in (third figure down: bidirectional arrows indicate filling-in within the completed boundaries), to yield a solid connected bar (fourth figure down) [Reprinted from Grossberg (2021)].

These gaps in retinal processing do not interfere with conscious vision because our eyes jiggle rapidly in their orbits, thereby creating transient signals from even stationary objects. These transients refresh the firing of retinal neurons. Because the blind spot and retinal veins are attached to the retina, they create no transients. Their inputs, therefore, fade, resulting in a retinal image where light is occluded at multiple positions (Figure 7).

The visual cortex uses multiple processing stages to complete boundaries and surfaces over these occluded positions. I call this process *hierarchical resolution of uncertainty* (Grossberg and Mingolla, 1985a,b; Grossberg, 1987a,b, 1994, 2021; Grossberg and Todorovic, 1988). Completed boundary and surface representations of mommy's form are computed in cortical area V4 of the What cortical stream (Grossberg, 1994, 1997, 2016; Grossberg and Pessoa, 1998; Kelly and Grossberg, 2000; Grossberg and Swaminathan, 2004).

**Figure 8:**
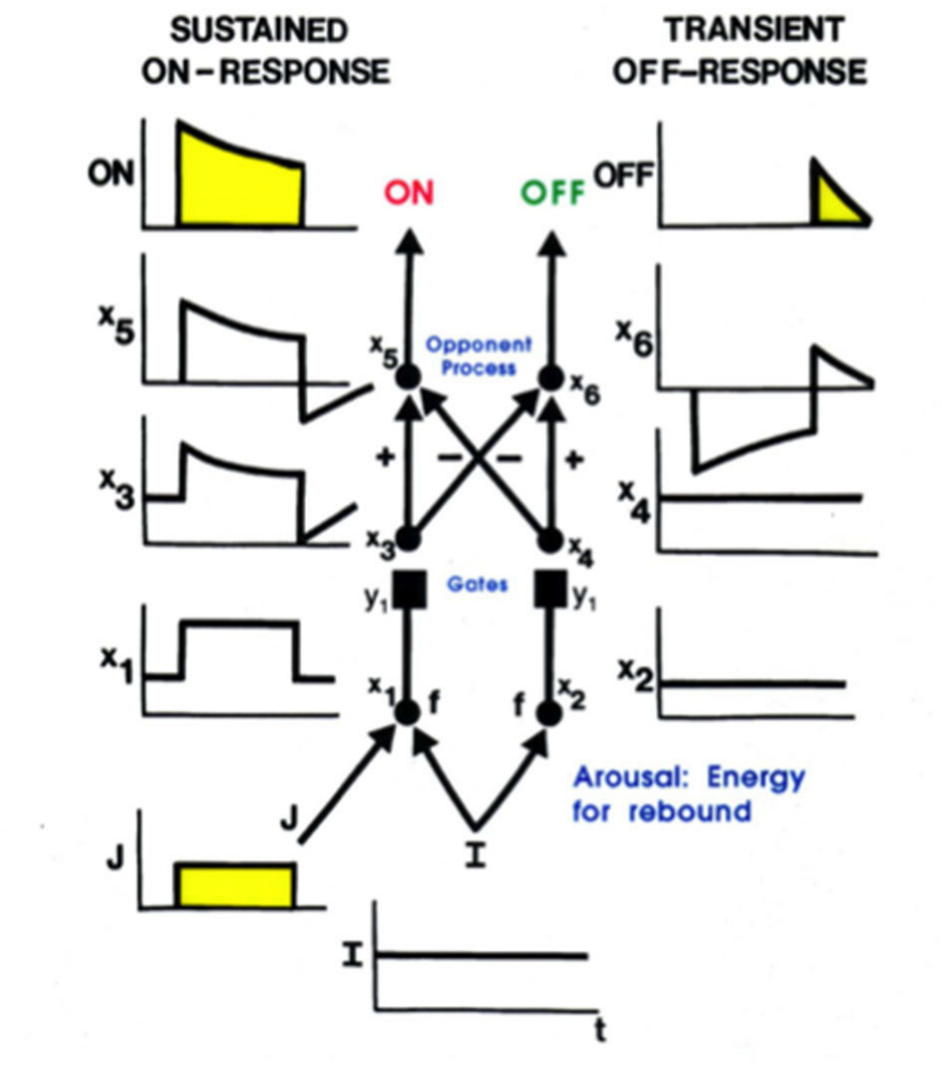
A gated dipole opponent process can generate a sustained habituative ON response in its ON channel in response to a steady input, J, to it, and a transient antagonistic rebound from its OFF channel in response to offset of J [Reprinted with permission from Grossberg (2021)].

Mommy's motion could produce streaks of persisting representations of her form at her earlier positions, much like a moving comet leaves a tail. Such persisting streaks could seriously degrade percepts of a scene, as well as recognition of mommy's form. Remarkably, these streaks are limited by the same mechanism that generates a representation of mommy's form, notably a *gated dipole* opponent process (Figure 8). A gated dipole ON channel selectively responds to the onset of mommy's image at its position(s). As mommy moves off a position, the gated dipole OFF channel at that position triggers a transient *antagonistic rebound* which inhibits the ON response to mommy, thereby limiting its persistence (Francis et al., 1994; Francis and Grossberg, 1996a,b; Grossberg, 2021).

## 8. Learning categories of mommy's movements: What-to-Where FORMOTION interactions

A series of changing positions of a moving form like mommy is computed in the What cortical stream. Perceiving a series of an object's changing positions is not, however, the same thing as perceiving its motion. Indeed, object motion is computed in the Where cortical stream.

Computations of form and motion are done in separate cortical streams because object form is sensitive to the *orientation* of an object's boundaries, whereas object motion is sensitive to an object's *direction* of motion. A good estimate of motion direction pools directional estimates from all an object's boundaries, across all their different orientations, that move in the same direction. A computation of motion direction hereby destroys the information that computes object orientation.

These parallel computations of object form and object motion are *computationally complementary* (Grossberg, 1991). The What stream uses representations of object form to learn categories whereby to recognize objects. The Where stream uses representations of object motion to navigate while avoiding obstacles in cluttered scenes and to track moving targets (Grossberg et al., 1999; Browning et al., 2009a,b; Elder et al., 2009; Grossberg, 2021).

The Where stream needs a complete visual representation of an object's form to successfully track it. Such a representation in the What stream is topographically mapped into the Where cortical stream whose dynamics can track its motion through time. The 3D FORMOTION model simulates how this happens (Berzhanskaya et al., 2007).

When a complex object like mommy walks or points, different parts of her body move with different directions and speeds. Leg movements while walking, and arm movements while pointing, are perceived relative to the *reference frame* of mommy's moving body that is tracked to keep her foveated. The MODE, or Motion DEcision, model (Grossberg and Pilly, 2008) explains how tracking happens (Figure 5). Grossberg et al. (2011a) and Grossberg (2021) further explain how mommy's body, and her arm and leg movements relative to it, are simultaneously perceived.

## 9. Peak shift and behavioral contrast: computing relative directions of moving parts and wholes

How did evolution discover how to compute object reference frames and the relative motion of object parts? An elementary neural property that occurs in multiple parts of the brain is used. It is called *peak shift and behavioral contrast*. This property occurs in all recurrent shunting on-center off-surround networks whose cells interact via Gaussian receptive fields when two inputs occur with the following properties.

**Figure 9:**
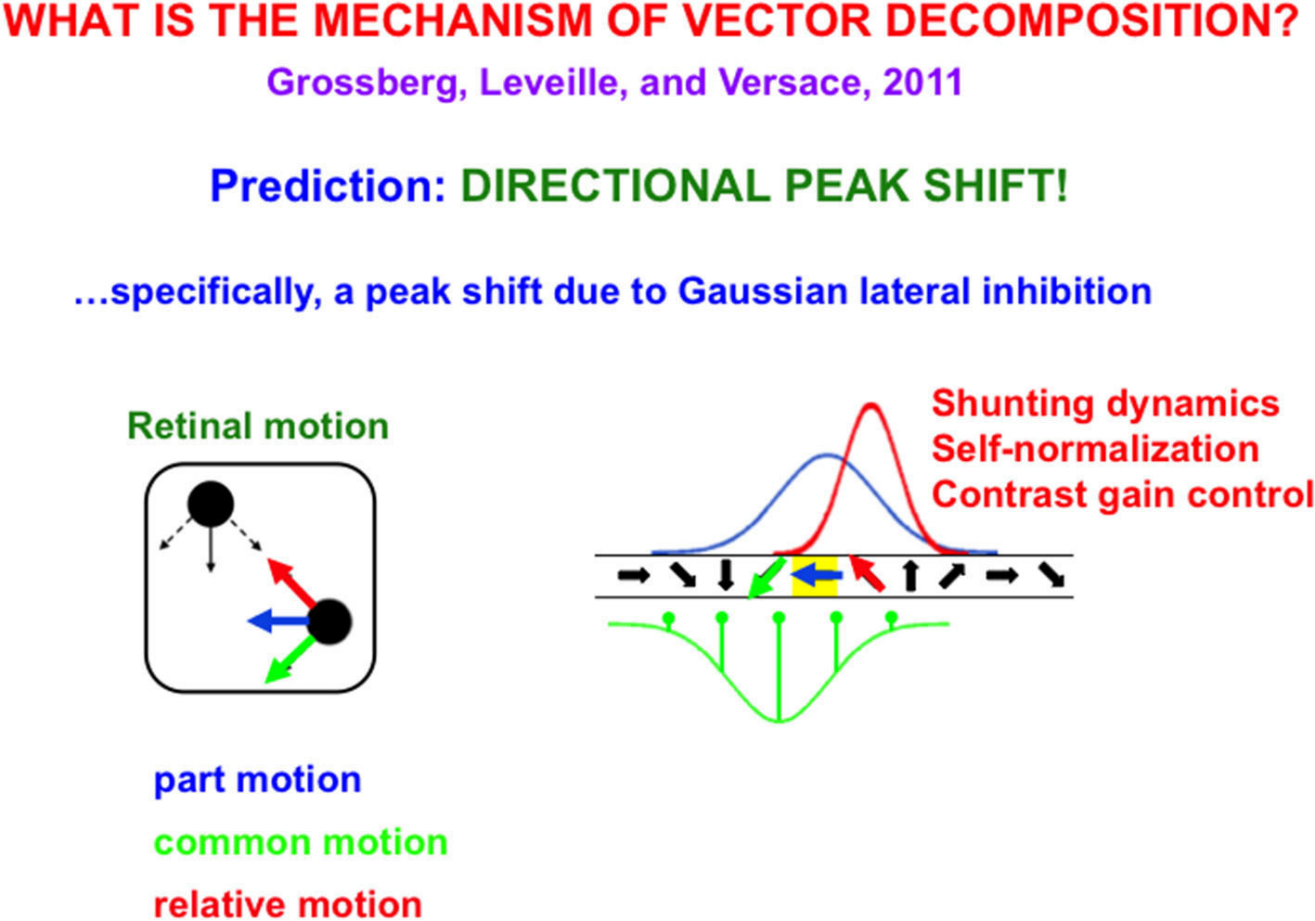
**(Left)** The upper left black dot moves up and down while the lower right black dot moves left (blue arrow) and right. Under proper spatial separations and motion speeds, the dots are perceived to be moving back and forth (red arrow) to and from each other, as their common motion moves diagonally downwards (green arrow). **(Right)** This motion illusion is due to a directional peak shift (red Gaussian) in a directional hypercolumn (spatially arrayed orientationally tuned cells). The common motion direction activates an inhibitory Gaussian receptive field (green), while the horizontal part of motion activates an excitatory Gaussian receptive field (blue) over the directional hypercolumn. When these inhibitory and excitatory Gaussians interact, the result is an excitatory Gaussian (red) whose maximal activity is shifted away from the inhibitory Gaussian (peak shift). Because interactions across this recurrent shunting on-center off-surround network are normalized, the red Gaussian, being narrower than the blue Gaussian, has higher maximal activity, thereby causing behavioral contrast [Reprinted with permission from Grossberg (2021)].

Suppose, in addition, that these neurons respond selectively to different *motion directions* in a topographic map (Figure 9), in particular, cells that are sensitive to oblique-upward motion directions occur between cells that are sensitive to upward and horizontal motion directions. Likewise, cells that are sensitive to oblique-downward motion directions occur between cells that are sensitive to downward and horizontal directions.

Motion directional inputs from mommy's body and her arm to such a network lead to a *vector decomposition* under the following conditions: suppose that the horizontal motion direction of mommy's body activates a horizontally tuned motion direction cell in the middle of its Gaussian on-center (Figure 9), while oblique motion directions of mommy's arm activate the Gaussian off-surround of the horizontal motion direction cell. When the oblique velocity vectors are subtracted from the horizontal velocity vector through time, the resulting velocity vectors generate oscillatory motion vectors of the arm's motion directions relative to the horizontally moving body.

Classical examples of such vector decompositions include the in-and-out motion direction percepts of dots relative to the moving frame that they define (Johansson, 1950) and rotational motion percepts of a wheel's spokes relative to the horizontal motion of its axel (Duncker, 1929/1938). Figure 9 shows how peak shift and behavioral contrast separate common motion direction from relative motion direction in response to a Johansson (1950) display. These percepts are explained and simulated in Grossberg et al. (2011b) and reviewed in Grossberg (2021).

As mommy walks, her leg that is further from the child is partly occluded to different degrees by the closer leg. A completed percept of the partially occluded leg is created and maintained using boundary and surface completion processes that realize *figure-ground separation* (Grossberg, 1994, 1997, 2016, 2021; Kelly and Grossberg, 2000). The completed representations can then be recognized while the child's brain computes their motion directions and speeds.

The *chopsticks illusion* of Anstis (1990) illustrates these properties using simpler stimuli; namely, a pair of rectangular black chopsticks moving in opposite directions on a white background, much as mommy's legs do.

## 10. Learning to say “mommy walks left” while observing her movements: nouns and verbs

How does a child's brain learn both a perceptual category and a language category for “walk” and “walking”? In particular, how is the perceptual representation of the *verb* in “mommy walk(s)” represented in the brain?

To do this, a view, or succession of views, of mommy standing up with her legs on the ground in a characteristic walking pose is classified, despite variations in perceptual features that differ across individuals who walk. Large receptive fields average across these details to extract mommy's overall shape and silhouette.

Multiple oriented scales, or filter sizes, from fine to coarse, initially process all incoming visual information. Higher-order processing stages select the scales that are most informative in different situations by associating them all with their predictive consequences. Only the informative scales will learn strong associations. Thus, as finer scales learn to categorize mommy's facial views, coarser scales learn to categorize actions like walking.

Suppose that the co-occurrence of two perceptual categories—of mommy's face and her walk pose—together trigger learning of a category that selectively fires when mommy walks, this *conjunctive* category can be associated via learning with the heard utterance “mommy walks” or “mommy is walking” via a bi-directional *associative map*.

A single pose of walking is often enough to recognize walking, just as a single pose of standing is enough to recognize that posture. Recognition of movements that cannot be effectively categorized using a single pose requires the Where cortical stream.

## 11. Learning to recognize movement direction and using it to track a moving target

**Figure 10:**
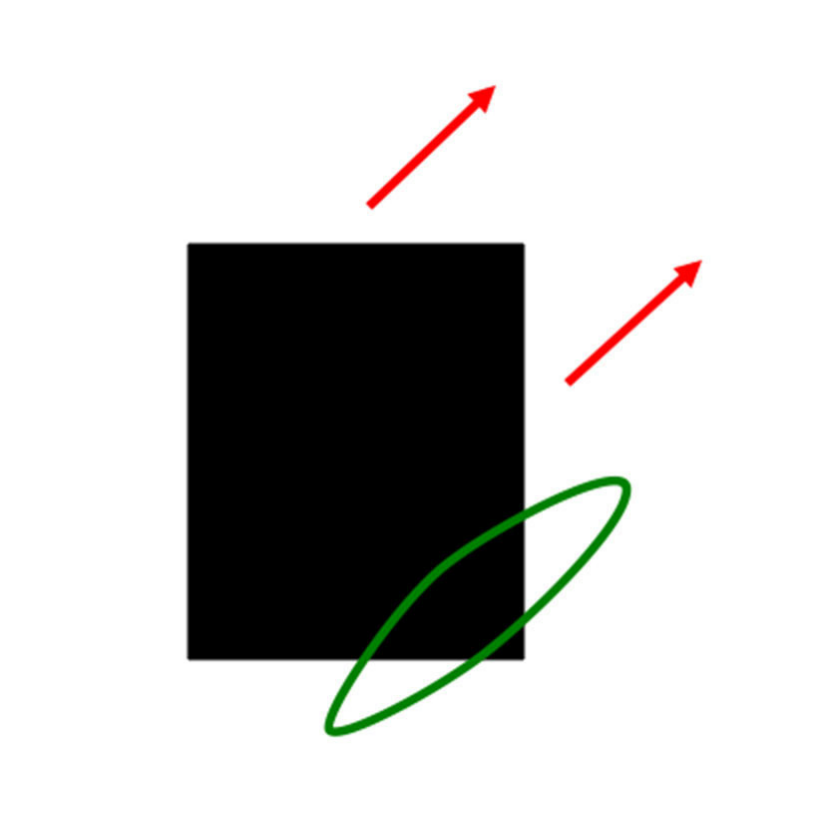
A directionally tuned motion-sensitive cell pools all possible contrast-sensitive sources of information that are moving in its preferred direction, and at similar motion directions. In this figure, the black rectangle is moving diagonally upwards. The directional motion cell (green ellipse) has a similar preferred motion direction. It pools diagonal motion signals from both the black-to-white vertical right-hand edge of the rectangle and the white-to-black horizontal bottom edge of the rectangle [Reprinted with permission from Grossberg (2021)].

As I noted in Section 5, interacting brain regions control eye movements that maintain the foveation of an attended moving object like mommy. Suppose that the linear motion of mommy's body activates a long-range directional filter. Such a filter has an elongated shape that adds inputs from an object's motion signals over time that move in its preferred direction when they cross its receptive field (Figure 10). Arrays of such filters exist in the Where cortical stream, each tuned to a different preferred direction (Albright, 1984; Rodman and Albright, 1987). These filters compete across directions at each position to choose the filter, or a nearby subset of filters, that is most activated by the moving object. They are a Where stream analog of the orientation columns in cortical area V1 of the What stream (Hubel and Wiesel, 1962, 1968, 1977).

Suppose that, when a directional filter is activated enough to fire for a sufficiently long time, its signals trigger learning of a directional motion category in the Where stream (Farivar, 2009). This category can then be associated, through learning, with a word or phrase in the What stream that best describes it, such as “left”.

When the perceptual categories for recognizing “mommy”, “walks”, and “left” are learned, they can be associated with a phrase like “mommy walks left” uttered by an adult speaker, as explained in Section 14.

## 12. Learning to say “mommy throws the ball” while observing her movements

How does a baby or child learn to say “Mommy throws ball” while observing mommy doing that? The first part of the sentence, “Mommy throws” can be understood in much the same way as “Mommy walks”. Suppose the child sees mommy in a profile view pull her arm back before thrusting it forward. Either extreme arm position may be sufficient to learn a category for “throw” in the What stream. The movement's motion can also be categorized in the Where stream.

If the arm that mommy uses to throw the ball is further away than her body, and thus partially occluded by her body during the movement, then a G-wave from the pulled-back location of her arm to its thrust forward position can flow “behind” her body, much as G-waves can flow behind scenic clutter when tracking a predator or prey (Grossberg, 1998, 2021).

As mommy completes the throw, a ball emerges from her hand and moves in the same direction. Often, attention will first focus on mommy's face. When the ball is thrown, attention flows to her arm, and then to the ball, via a G-wave. This temporal sequence of recognition events is thus: mommy, throws, ball. Perceptual categories that correspond to these events are activated and stored in a *perceptual working memory* in their correct temporal order, and trigger learning of a sequence category, or *list chunk*. A heard sentence category of “mommy throws ball” can be stored in a *linguistic working memory*, and trigger learning of its own list chunk. The linguistic list chunk learns an association to the perceptual list chunk, and conversely. These list chunks also send learned top-down signals to the working memory patterns that they categorize, which can be read out from working memory in the correct order, under volitional control by a GO signal from the basal ganglia. Seeing these events can hereby elicit a descriptive sentence (see Sections 14 and 15 for details).

## 13. Learning to say “watch mommy throw the ball” before or as she does so: nouns and verbs

A sentence such as “watch mommy throw the ball” or “Look at mommy throw the ball” can prime a child to orient to the mommy to experience her throwing the ball. Such priming is based on previous learning experiences.

First, a baby or child learns the meaning of the command to “Watch” or “Look” from a teacher who points in the direction that the baby or child should look while one of these words is uttered. Learning this skill required the social cognitive ability to share *joint attention* between the pointing teacher and the student. How our brains accomplish this feat was discussed in Section 5. The learner's attention can hereby shift to foveate mommy in response to the word “Watch” or “Look” while a teacher looks at and points to mommy.

The teacher may also utter the word “mommy” to form the sentence “Watch mommy”. How is the meaning of the sentence “watch mommy” learned? The words “Watch” and “mommy” get stored in working memory in the correct temporal order, and then trigger learning of a list chunk. As this is occurring, the baby or child is orienting toward mommy's face.

The list chunk can learn to activate an invariant face category of mommy, which reads out priming signals that subliminally activate all the view-selective and position-selective categories of mommy's face. The view category that codes the view and position in the visual field where mommy's face is observed will be activated for suprathreshold activities. Because of its position-selectivity, this view category in the What cortical stream can then read out a motor priming command to the corresponding positional representation of mommy's face in the Where cortical stream via a What-to-Where interaction.

When the position of mommy's face is primed, activation of the list chunk that codes “watch mommy” can also activate a non-specific GO signal from the basal ganglia to the Where cortical stream. Taken together, the positional prime and the GO signal fully activate the primed position and thereby elicit orienting movements to foveate mommy.

With this background, we can ask what parts of the brain are used to store and understand the sentence “Watch mommy throw the ball”? For starters, note that the meanings of the *verbs* “watch” and “throw” are understood by using cortical representations in the Where stream. In contrast, the meanings of *nouns* such as “mommy” and “ball” are understood by activating cortical representations in the What, cortical stream. Both noun and verb word representations are stored in the temporal and prefrontal cortices.

Thus, understanding the meaning of the sentence “Watch mommy throw the ball” requires alternate switching between the noun and verb representations in the What and Where cortical streams, respectively. Words like the article “the” that helps to structure sentences are part of *syntactics* and are discussed in Section 16.

In contrast, the branch of linguistics that is concerned with meaning is called *semantics*. Unlike the present article, traditional semantic studies do not link the language that embodies meanings with the real-word perceptual and affective experiences that they denote during language learning, e.g., Jackendoff (2006).

## 14. Temporary storage of linguistic items in working memory and their learned list chunks

Before continuing with our analysis of how a baby or child can learn language meanings, I will review some basic concepts about what working memories are and how they work. Working memories, and models thereof, temporarily store sequences of items or events as *item chunks*. An *item chunk* responds selectively when the distributed features that it represents are presented (e.g., a phoneme or musical note). Every time that we attentively listen to a sufficiently short series of words, they can be stored in a linguistic working memory, like the sentence: “Watch mommy throw the ball”.

If certain sequences of words are repeated often enough, they can be learned by a process that is alternately called *unitization* or *chunking*. If a series of words can be unitized into a single chunk that is called a *list chunk*, it selectively responds to prescribed sequences of item chunks that are stored in working memory (e.g., a word or the lyrics of a familiar song). These processes occur in brain regions like the ventrolateral prefrontal cortex (VLPFC) and dorsolateral prefrontal cortex (DLPFC).

**Figure 11:**
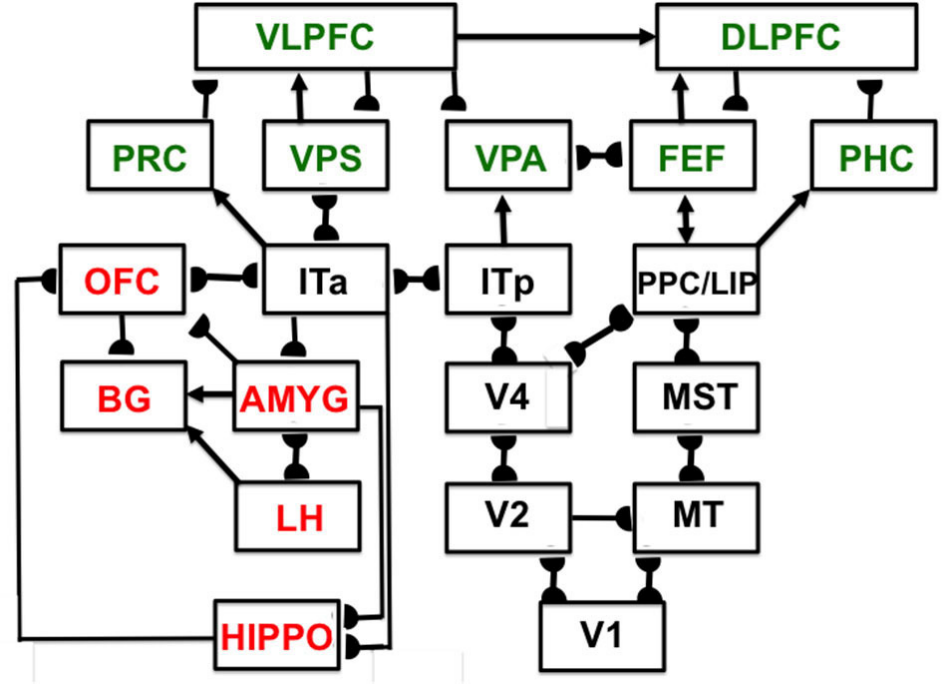
Macrocircuit of the main brain regions, and connections between them, that are modeled in the *predictive Adaptive Resonance Theory* (pART) of cognitive-emotional and cognitive working memory dynamics. Abbreviations in red denote brain regions used in cognitive-emotional dynamics and those in green denote brain regions used in working memory dynamics. Black abbreviations denote brain regions that carry out visual perception, learning, and recognition of visual object categories, and motion perception, spatial representation, and target tracking. Arrows denote non-adaptive excitatory synapses. Hemidiscs denote adaptive excitatory synapses. Many adaptive synapses are bidirectional, thereby supporting synchronous resonant dynamics among multiple cortical regions. The output signals from the basal ganglia that regulate reinforcement learning and gating of multiple cortical areas are not shown. Also not shown are output signals from cortical areas to motor responses. V1, striate, or primary, visual cortex; V2 and V4, areas of prestriate visual cortex; MT, middle temporal cortex; MST, medial superior temporal area; ITp, posterior inferotemporal cortex; ITa, anterior inferotemporal cortex; PPC, posterior parietal cortex; LIP, lateral intraparietal area; VPA, ventral prearcuate gyrus; FEF, frontal eye fields; PHC, parahippocampal cortex; DLPFC, dorsolateral hippocampal cortex; HIPPO, hippocampus; LH, lateral hypothalamus; BG, basal ganglia; AMGY, amygdala; OFC, orbitofrontal cortex; PRC, perirhinal cortex; VPS, ventral bank of the principal sulcus; VLPFC, ventrolateral prefrontal cortex [Reprinted with permission from Grossberg (2021)].

Interactions of list chunks with other brain regions, including perirhinal cortex (PRC), parahippocampal cortex (PHC), amygdala (AMYG), lateral hypothalamus (LH), hippocampus (HIPPO), and basal ganglia (BG), enable predictions and actions to be chosen that are most likely to succeed based on sequences of previously rewarded experiences. Figure 11 summarizes a model macrocircuit of the predictive Adaptive Resonance Theory, or pART, model of cognitive and cognitive-emotional dynamics that explains how these interactions work. The seven relevant prefrontal regions that are involved in storing, learning, and planning sequences of events are colored green in Figure 11.

**Figure 12:**
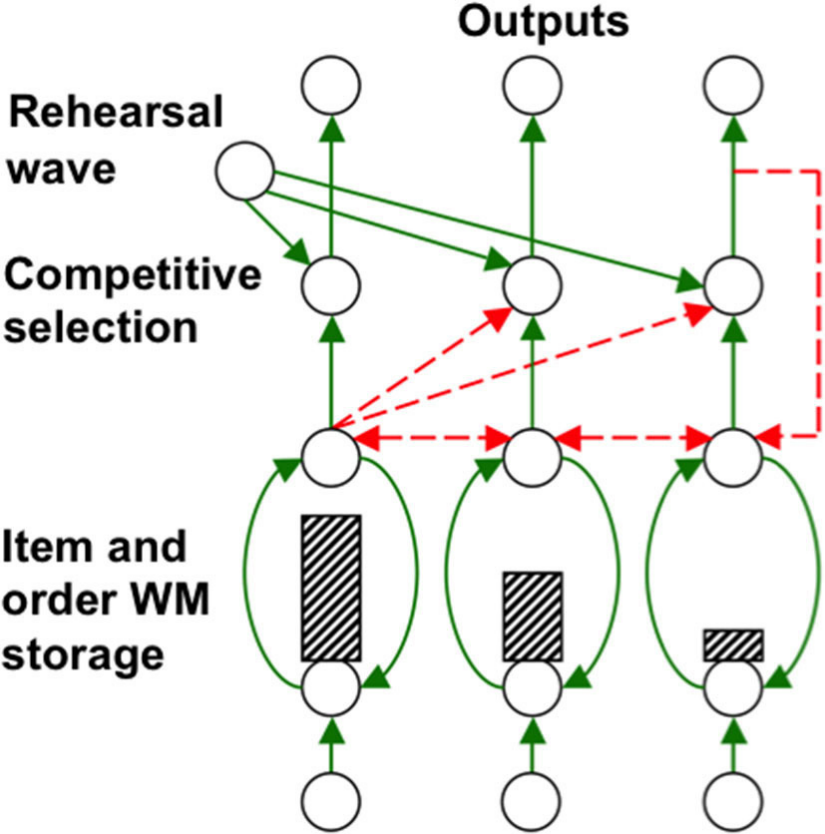
Item-and-Order working memory model: Primacy gradient of activity (hatched vertical rectangles) stored in working memory within a recurrent shunting on-center (vertical curved green recurrent arrows) off-surround (horizontal recurrent dashed red arrows) network. Rehearsal is controlled by a non-specific rehearsal wave (diverging green diagonal arrows) followed by self-inhibitory feedback of the item that is currently being rehearsed (vertical dashed red arrow) [Reprinted with permission from Grossberg (2021)].

Grossberg (1978a,b) introduced a neural model of working memory, which has been incrementally developed to the present time. In it, a sequence of inputs that occurs one at a time is stored as an evolving *spatial pattern* of activation of *item chunks* that code the cell populations of the working memory (Figure 12). This working memory model is called an Item-and-Order model because individual cell populations represent list *items*, and their temporal *order* of occurrence is stored by an *activity gradient* across the populations (Figure 12).

A generalization of this model, called the Item-Order-Rank, or IOR, model, enables lists of items to be stored in which some of the items are repeated, such as repeated letters, as in the sequence “ABACBD”, or repeated words, as in “my true love is true” (Bradski et al., 1994; Grossberg and Pearson, 2008; Silver et al., 2011; Grossberg, 2018, 2022). Other kinds of events can also be temporarily stored in working memory, such as the turns taken during navigation to a goal, the arm movements made during a dance, or the notes played in a musical melody. Remarkably, a *single* canonical circuit design, suitably specialized, can store auditory, linguistic, spatial, or motor sequences in multiple working memories that operate in parallel in the prefrontal cortex.

Why should we believe that IOR working memories exist in the brain? As reviewed elsewhere (Grossberg, 2021, 2022), they provide unified and principled explanations of many psychological and neurobiological data about working memory and list chunk dynamics. No less important is the fact that they explain why and how sequences of items and events that are stored in working memory are learned and stably remembered through time as list chunks.

Item-and-Order working memories are a unique kind of circuits that embody two simple postulates that enable their list chunks to be learned and stably remembered: the *LTM Invariance Principle* and the *Normalization Rule*. These postulates were used to derive mathematical equations for Item-and-Order working memories when they were first introduced by Grossberg (1978a,b).

The LTM Invariance Principle prevents the storage of longer lists of events in working memory (such as MYSELF) from causing catastrophic forgetting of previously learned list chunks of its shorter sublists (such as MY, SELF, and ELF). It guarantees that, if bottom-up inputs lead to storage of a familiar list chunk, say for the word MY, then storing the word SELF to complete storage of the novel word MYSELF will not cause forgetting of the learned weights that activated the list chunk of MY.

The Normalization Rule just says that the *maximum total activity* that is stored across a working memory is independent of the number of activated cells. This Rule follows from the fact that the cells in an Item-and-Order working memory compete among themselves via a *recurrent shunting on-center off-surround network* (Figure 12; Section 7). Such networks occur ubiquitously in our brains because they solve what I have called the *noise-saturation dilemma* (Grossberg, 1973, 2021). Simply put, the cells in such a network can store the relative sizes, and thus importance, of inputs in their activities without flattening the pattern of activity with saturation, or obscuring them in cellular noise.

The LTM Invariance Principle and Normalization Rule also imply that only short lists can be stored in working memory in a way that enables their performance in the correct temporal order. In particular, a stored *primacy gradient* of activity enables items to be recalled in the correct temporal order. In a primacy gradient, the first item in the sequence is stored in working memory with the most activity, the second item in the sequence activates its item chunk with the next largest activity, and so on, until all items are stored (Figure 12). For example, the primacy gradient that stores a sequence “A-B-C” of items stores “A” with the highest activity, “B” with the second highest activity, and “C” with the least activity.

A stored *spatial* pattern in working memory is *recalled* as a *temporal* sequence of items when a *rehearsal wave* uniformly, or non-specifically, activates all the working memory cells (Figure 12). This rehearsal wave is emitted by the basal ganglia, or BG (Figure 11). The cell population with the highest activity is read out fastest because it exceeds its output threshold fastest. While it is read out, it self-inhibits its working memory representation via a recurrent inhibitory interneuron (Figure 12), a process that is called the *inhibition-of-return* in the cognitive science literature (Posner et al., 1985). Inhibition-of-return prevents the perseverative performance of the most recent item. Each rehearsed item representation self-inhibits, therefore enabling the cell population with the next largest activity to be read out until the entire sequence is performed. Much data about working memory dynamics are explained and quantitatively simulated using an Item-and-Order model by Grossberg and Pearson (2008) and reviewed by Grossberg (2021).

The flexibility of working memory makes it possible to assemble novel sentences on the fly, since all that matters, after multiple sources may input to the working memory, is the final *activity gradient* that determines the temporal order in which the words in a phrase are performed. Phrases that are stored using a *primacy gradient* can then be performed in the order that they are loaded into working memory. As in musical performance, such a grouping, or phrase, is stored before being unitized into list chunks that enable the automatic future performance of whole sequences of words.

Just three interacting processing levels are sufficient to store, learn, and perform long sequences of items or events that include repeats, such as in the lyric “our true love was true”. Our brains do not need, nor do they have, many processing levels to store, learn, and perform sequential behaviors. Figure 11 illustrates that each brain region carries out different tasks with its own characteristic anatomy, in contrast to Deep Learning models that may include over 100 networks in a hierarchy, each with similar connectivity (Srivastava et al., 2015).

## 15. The basal ganglia gate cortical representations of percepts, concepts, feelings, and actions

All parts of our brain, including cognitive and motor working memories, are gated ON and OFF by the basal ganglia, which are part of multiple feedback loops running through all cortical representations of percepts, concepts, feelings, and actions (Hikosaka and Wurtz, 1983, 1989; Alexander et al., 1986; Alexander and Crutcher, 1990; Brown et al., 1999, 2004; Middleton and Strick, 2000; Dubois et al., 2007; Grahn et al., 2009; Silver et al., 2011; Grossberg, 2016, 2021). For example, in the control of planned sequences of saccadic eye movements, three different basal ganglia loops are used to store, choose, and perform saccades in the movement sequence. They are modeled by Silver et al. (2011) and reviewed by Grossberg (2021).

The volitional GO signal that has been mentioned in several places above is an example of basal ganglia gating. Indeed, all brain representations are gated ON and OFF by one or another basal ganglia loop.

## 16. Learning definite and indefinite articles and how to use them in sentences

The sentences that have been considered so far have not included definite or indefinite adjectives, also called *articles* (De Villiers and Villiers, 1973). The full richness of English language meanings cannot, however, be understood without articles. To start, let us compare the meanings of the indefinite article “a” and the definite article “the” in the phrases “a ball” and “the ball”.

The sentence “It is a ball” could refer to any ball. In contrast, the sentence “Watch the ball” refers to a particular ball. The following quote about *Definite and Indefinite articles*, from *Study.com*, explains this distinction in greater detail.

“An article” is a word used to modify a noun, which is a person, place, object, or idea. Technically, an article is an adjective, which is any word that modifies a noun. Usually, adjectives modify nouns through description, but articles are used instead to point out or refer to nouns. There are two different types of articles that we use in writing and conversation to point out or refer to a noun or group of nouns: definite and indefinite articles.

“The definite article (the) is used before a noun to indicate that the identity of the noun is known to the reader. The indefinite article (a, an) is used before a noun that is general or when its identity is not known. There are certain situations in which a noun takes no article”.

## 17. Using definite and indefinite articles with nouns and verbs

Consider the variations: “It is the ball” or “That is the ball” vs. “It is a ball” or “That is a ball”, or the analogous sentences “Watch the ball” vs. “Watch a ball”. The word “is” may be ambiguous in more than one sense: It can precede a *noun* (object word) or a *verb* (action word). For example, the phrase “is a” disambiguates “is” to precede a noun. In contrast, “is throwing” illustrates how “is” can precede a verb. You can say that “Mommy is throwing the ball” or “Mommy is throwing a ball” depending on whether a particular ball is intended.

How does a baby learn the difference between “Mommy throws a ball” and “Mommy is throwing a ball”? Or the difference between “Mommy throws the ball” and “Mommy is throwing the ball”?

Both kinds of sentences can refer to the same action. Replacing “throws” with “is throwing” can emphasize that the action is occurring now, and can be learned from a teacher while witnessing the event.

## 18. Learning to separate articles from nouns: attentional blocking and unblocking

How does the child's brain learn to translate the experience of *seeing* a/the ball being thrown into *saying* a verbal phrase like “a/the ball is being thrown”? Or saying a verbal phrase like “a big ball” or “the green ball” is being thrown?

A number of scientific studies have used various kinds of functional neuroimaging to study the brain activity patterns that are elicited by adjective-noun phrases (e.g., Chang et al., 2009; Lange et al., 2015; Fyshe et al., 2016, 2019; Kochari et al., 2021). These studies, as well as other studies of how adjectives interact with nouns (e.g., Sandhofer and Smith, 2007) do not, however, describe how adjective-noun phrases interact with the perceptual representations that embody their meaning in the world.

For example, Fyshe et al.'s (2016, p. 4458) wrote: “The computational (rather than neuroscientific) study of language semantics has been greatly influenced by the idea that word meaning can be inferred by the context surrounding a given word, averaged over many examples of the word's usage…. For example, we might see the word ball with verbs like kick, throw, and catch, with adjectives like bouncy or with nouns like save and goal. Context cues suggest meaning, so we can use large collections of text to compile statistics about word usage (e.g., the frequency of pairs of words), to build models of word meaning.” Although “context cues suggest meaning”, they only do so because humans who use these cues can link them to the perceptual representations that they reference.

In particular, are the phrases ”a ball” or “the ball” learned as *list chunks*, or unitized representations, in their own right when a child listens to mommy speak about a perceptual event that involves a ball? How, then, are articles ever dissociated from the particular nouns with which they co-occur?

**Figure 13:**
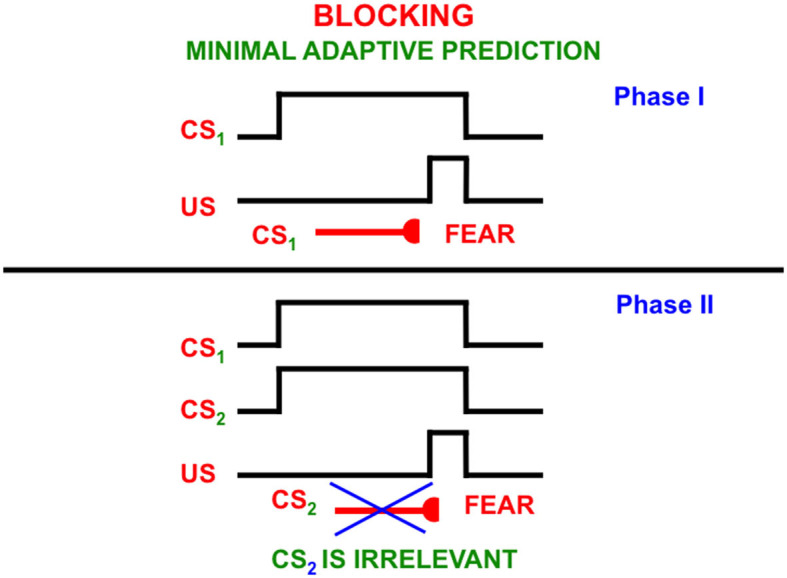
The blocking paradigm illustrates how cues that do not predict different consequences may fail to be attended. During the training Phase I, a conditioned stimulus (CS_1_) occurs shortly before an unconditioned stimulus (US), such as a shock, on enough learning trials for CS_1_ to become a conditioned reinforcer whose activation elicits conditioned fear. During Phase II, CS_1_ is presented simultaneously with an equally salient, but unconditioned stimulus, CS_2_, the same amount of time before the same unconditioned stimulus. This can be done on quite a few learning trials. Despite this training protocol, CS_2_ does not learn to elicit fear. It has been *attentionally blocked* by CS_1_ [Reprinted with permission from Grossberg (2021)].

This can be explained by processes such as *attentional blocking* (Pavlov, 1927; Kamin, 1968, 1969; Grossberg, 1975, 2018; Grossberg and Levine, 1987; Grossberg and Schmajuk, 1989; Grossberg and Merrill, 1992, 1996). Attentional blocking of a word or perceptual object can occur when it is predictively irrelevant. It is then suppressed and not attended (Figure 13). *Unblocking* of a suppressed word or object can occur when it becomes predictively relevant again.

Since the word “ball” is always associated with the perceptual experience of a ball, it is predictively relevant in phrases like “a ball” and “the ball”. But the articles “a” and “the” are not, at least at first. Rather, they may co-occur with many other words and are chosen via a one-to-many mapping from each article to the many words with which it co-occurs in sentences. When the articles are suppressed, the primacy gradient that stores the word “ball” in working memory can trigger learning of a list chunk of the word that can be associated with visual categories of the perceptual experiences of seeing a/the ball.

An article can remain predictively irrelevant and blocked until a predictive perceptual context, and thus a language meaning, is associated with a phrase like “a ball”, when an unfamiliar ball is experienced, or “the ball” when the ball is a particular or familiar one. In these situations, the phrases “a ball” and “the ball” in working memory may trigger learning of their own list chunks.

Behavioral interactions between a teacher and a learner like the following may help to understand how the meanings of these phrases are learned: suppose that a child says “Mommy throws ball”, and mommy says in return “This is *the* ball daddy bought”. If experiences like this happen enough, the child can learn that “*the* ball” may refer to a particular or familiar ball and, as noted above, the phrase “the ball” may be learned as a list chunk in response to its recurring representation as a primacy gradient in working memory.

Keep in mind that the articles interact with both perceptual and cognitive processes: on the perceptual side, the choice of which article “a” or “the” to store in working memory depends upon first perceiving, or at least imagining, the object that the article modifies. The article “the” may refer to a particular, or familiar, ball, as in the sentence: “Mommy threw the ball”. The article “a” may refer to any ball, especially an unfamiliar one, as in the sentence: “Pick a ball from the pile.” On the cognitive side, the appropriate articles are inserted into phrases and sentences that are stored in a linguistic working memory, along with the nouns that they modify, before the stored items are performed in response to a volitional GO signal.

Adjectives and adverbs can influence what is perceived when constructing a sentence or imagined when hearing the sentence, e.g., “big ball”, “quickly running”, etc. Adjective-noun and adverb-verb phrases can also correspond to experienced percepts and hearing them can trigger visual, or other perceptual, memories of such experiences. These words can be inserted in sentences in much the same way as articles are.

Consistent with this analysis, Chang et al. (2009) note: “Multiplicative composition models of the two-word phrase outperform additive models, consistent with the assumption that people use adjectives to modify the meaning of the noun, rather than conjoining the meaning of the adjective and noun.” This type of result indicates that adjectives are bound together with their nouns to modify their meaning, but does not clarify how such phrases learn to represent that meaning in the first place.

## 19. Learning to associate visual objects with their auditory names

To know where in the brain, and how, an article like “a” or “the” is inserted into a phrase or sentence, more detail is needed about the processes whereby visual events like objects are unitized through learning into object categories. These visual categories are then associated with their learned auditory names in working memory. Said in another way, a noun's *name* can be learned when it is associated through visual-to-auditory learning with a learned visual category of the object that it represents (e.g., a ball). Biological neural networks that are capable of such learned mappings are described in greater detail below.

As this mapping is described in more detail below, it will become clear that the ability to fluently recognize an object as a ball does not, in itself, determine whether the name “ball” should be modified by the article “a” (“That's a ball”) or “the” (“That's the only ball that I have”). It is often not the ball-like features that determine article choice about a previously learned object. Rather, it is whether a particular combination of these ball-characterizing features is *familiar*, say due to a particular texture or design on it, or an unusual size-color combination, and the like; or whether an object that is an exemplar of a previously learned category is being used in a definite context, e.g., “Watch mommy throw the ball”.

To understand how these properties arise, let us review, and ground in different brain regions, some of the steps that go into learning an object's visual recognition category, whether it is for a ball, mommy's face, or whatever: as noted in Section 7, the functional units of 3D vision are perceptual boundaries and surfaces. The visual boundaries and surfaces that are, for example, used to perceive a ball are computed in the striate and prestriate visual cortices, including cortical areas V1, V2, and V4, in the What stream (Motter, 1993; Sereno et al., 1995; Gegenfurtner, 2003).

After the processing of visual boundaries and surfaces is complete (e.g., in cortical area V4), they are then categorized at subsequent cortical processing stages. As noted in Section 3, a particular view of a surface like mommy's face can be learned and recognized by a category within the posterior inferotemporal cortex, or ITp. An invariant category that selectively responds to multiple views of mommy's face can be learned within the anterior inferotemporal cortex, or ITa. Such an invariant category may reciprocally interact via bi-directional adaptive connections with all the view categories of mommy's face in ITp (Figure 2A; for further discussion and supportive data, see Grossberg, 2021).

These visual object recognition categories, in turn, activate additional processes at higher cortical areas, such as those that code familiarity about objects, including the anterior temporal cortex, anterior occipitotemporal sulcus, anterior fusiform gyrus, posterior superior temporal sulcus, and the precentral gyrus over the frontal cortex (Chao et al., 1999; Bar et al., 2001; Haxby et al., 2001; Rajimehr et al., 2009; Sugiura et al., 2011; Huth et al., 2012; Bonner and Price, 2013; Ramon and Gobbini, 2018; Kovács, 2020). Auditory object name categories, and facts about them, maybe computed in the anterior temporal cortex, among other cortical areas (Hamberger et al., 2005; Bemis and Pylkkänen, 2013).

**Figure 14:**
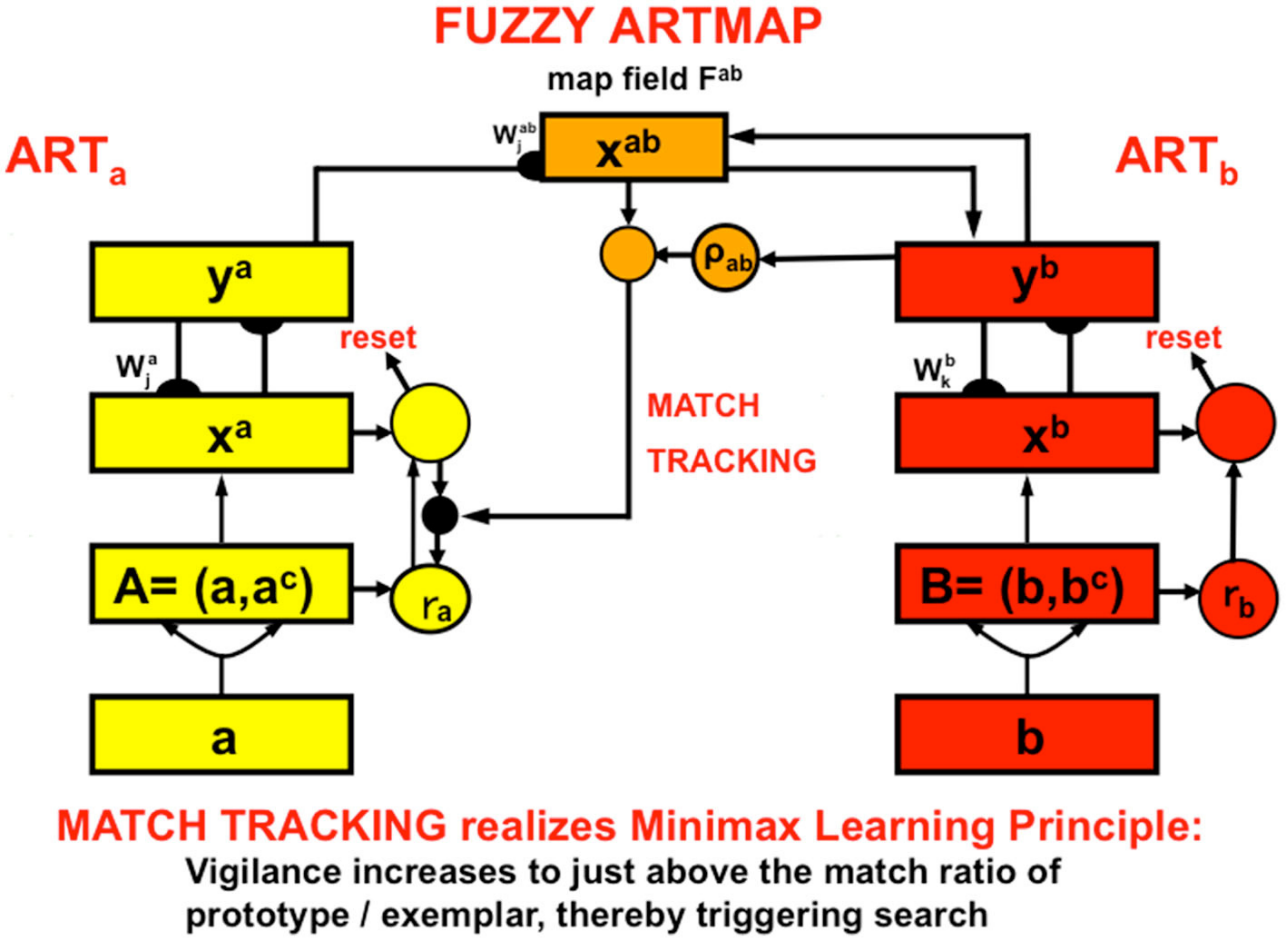
A system like Fuzzy ARTMAP can learn to associate learned categories in one ART network (yellow processing stages) with learned categories in a second ART network (red processing stages). Because both bottom-up and top-down interactions occur in both networks, a bottom-up input pattern to the first ART network (ARTa) can learn to generate a top-down output pattern from the second ART network (ARTb). In this way, an input pattern to ARTa can elicit a predicted output from ARTb [Reprinted with permission from Grossberg (2021)].

Visual recognition categories and auditory name categories can be linked through learning by an associative map. Neural network models of intermodality map learning include ARTMAP, for learning binary mappings; and fuzzy ARTMAP for learning binary or analog mappings, among other variants (Figure 14) (Carpenter et al., 1991, 1992, 1995, 1997, 1998, 2005; Asfour et al., 1993; Bradski and Grossberg, 1995; Carpenter, 1997, 2003; Grossberg and Williamson, 1999; Granger et al., 2000; Carpenter and Ravindran, 2008).

**Figure 15:**
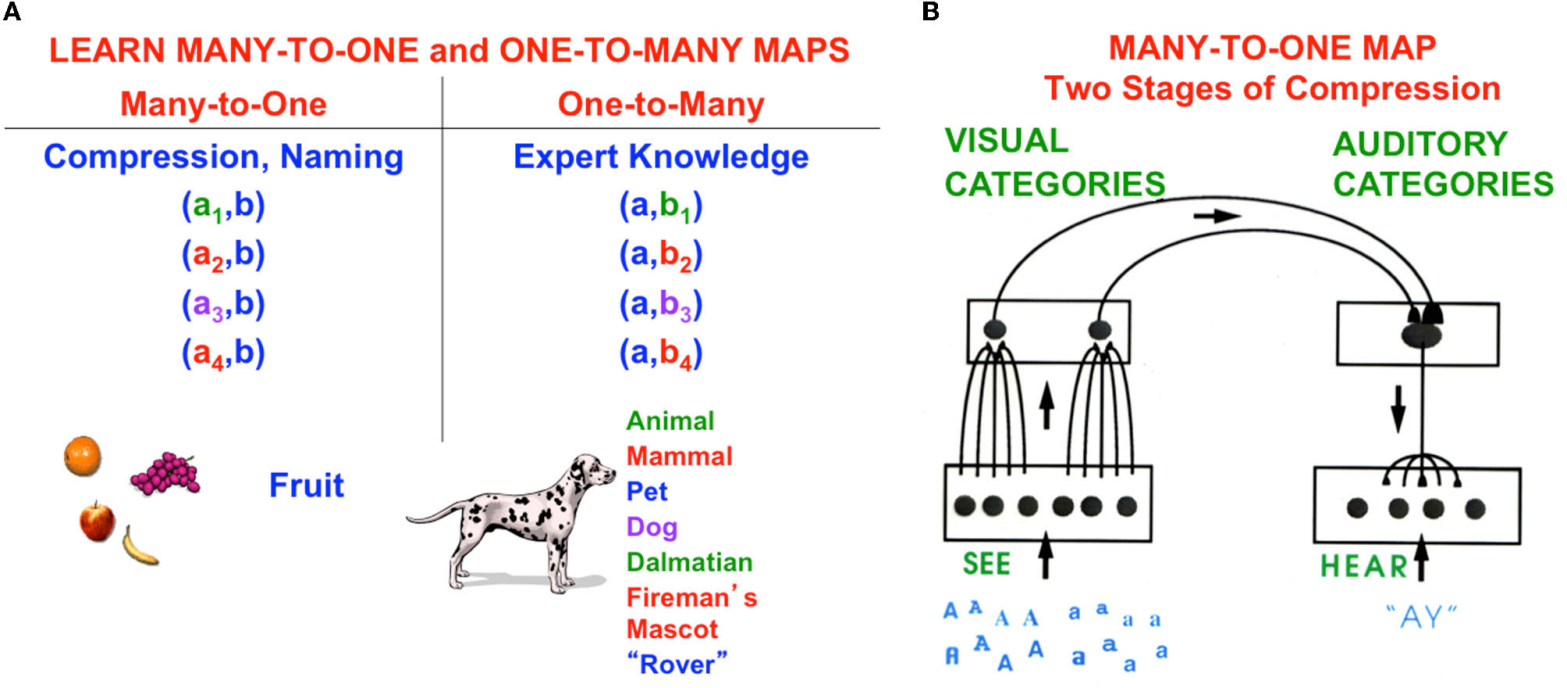
**(A)** Humans and other autonomous adaptive intelligent agents need to be able to learn both many-to-one maps and one-to-many maps. **(B)** Learning a many-to-one map from multiple visual fonts of a letter to the letter's name requires a stage of visual category learning that is linked to a stage of auditory name learning via a learned associative map. Figure 14 shows that a Map Field is needed to link learned visual categories with learned auditory categories via associative learning [Reprinted with permission from Grossberg (2021)].

Figure 15A depicts two different kinds of associative maps: Many-to-one maps and one-to-many maps. The example in Figure 15A of a many-to-one map shows how visual images of multiple different kinds of fruit are mapped into the same name “fruit”. The example of a one-to-many map in Figure 15B shows how a single image of a dog can be associated with many different words to describe the dog, ranging from the general word “animal” to the specific name of this particular dog “Rover”. As more names are associated with the dog's image, our “knowledge” about the dog expands.

Figure 15B illustrates that learning a many-to-one map requires two different stages of learning. In this example of visual-to-auditory learning, multiple fonts of a letter A drive the learning of multiple visual categories that selectively respond to variations of each letter font. Because the letter fonts A and a are composed of such different visual features, they learn to activate different visual categories. Then, these visual categories are all associated with the same auditory name of the letter via a Map Field (Figure 14).

## 20. Map fields are working memories

The discussions above have clarified how observing a visually experienced sequence of events during which mommy throws a ball can lead to that sequence of events being stored, in the same order, in linguistic working memory as a sequence of words describing that sequence. The sentence “mommy throws the ball” was mentioned as one example. Putting together the discussions of working memories and Map Fields leads to the conclusion that a Map Field is also a working memory where linguistic sequences can be stored in response to sequential activation of their visual categories through time. It is also possible that a Map Field topographically inputs to a working memory, but that the Map Field itself does not have the recurrent interactions or GO signal modulation of a working memory.

How to think of the choice of articles “a” or “the” in such sequences depends upon whether they are in response to hearing speech that is uttered by someone else, as in a sentence like “Watch mommy throw the ball”, or self-generated speech in response to an externally viewed, or internally remembered, perceptual experience like “Mommy threw a ball”. Since children learn at least their first languages by listening to teachers who know the language, the choice of the article will depend on the perceptual experiences to which the teachers' utterances correspond.

## 21. Resonance between bottom-up adaptive filters and top-down learned expectations: ART

Figure 15B shows only bottom-up adaptive pathways between the distributed feature pattern of each letter and its visual category. In the brain, as well as in Adaptive Resonance Theory, or ART, models of object category learning, there are both bottom-up and top-down adaptive pathways (Figures 2, 11, 14). The bottom-up pathways form an *adaptive filter* because the ends of these pathways contain adaptive weights, or long-term memory (LTM) traces, that can learn the feature patterns that characterize each font category. The top-down pathways activate a prototype, or expectation, that can also learn the same feature pattern. This feature pattern is called a *critical feature pattern* because it includes only those features that past learning has shown to control learning and correct predictions. Outlier features are suppressed during learning because they are predictively irrelevant.

When both bottom-up and top-down pathways are simultaneously active, the signals that they read out synchronize, amplify, and focus attention on the critical feature pattern that reliably codes the correct category. The synchronous resonance between features and categories also triggers fast learning within the bottom-up and top-down adaptive weights that lead to and from the currently active category. That is why the resonance is called an *adaptive* resonance. No less important, top-down matching by a learned expectation protects the learned adaptive weights from being destabilized by catastrophic forgetting. They hereby solve the fundamental *stability-plasticity dilemma*. Namely, they support fast learning (plasticity) while protecting these learned weights from experiencing catastrophic forgetting (stability) (see Grossberg, 2021 for further details).

## 22. Concluding remarks: “the limits of my language mean the limits of my world”

The famous quote in this section's header is by Ludwig Wittgenstein in his classic *Tractatus Logigo-Philosophicus* (Wittgenstein, 1922). This simple sentence summarizes both the main contribution of the present article and its main limitation: The main contribution is to create a theoretical foundation for understanding how humans learn in real time to link language utterances to real-world experiences that give them meaning. The main limitation is that the open-ended nature of language enables it to bridge “the limits of [our] world”. Providing an analysis of how we learn all the language meanings that we can express about our world will require many scientists working for years to complete.

## Data availability statement

The original contributions presented in the study are included in the article, further inquiries can be directed to the corresponding author.

## Author contributions

The author confirms being the sole contributor of this work and has approved it for publication.
